# Commercial scale genetic transformation of mature seed embryo explants in maize

**DOI:** 10.3389/fpls.2022.1056190

**Published:** 2022-11-29

**Authors:** Xudong Ye, Ashok Shrawat, Edward Williams, Anatoly Rivlin, Zarir Vaghchhipawala, Lorena Moeller, Jennifer Kumpf, Shubha Subbarao, Brian Martinell, Charles Armstrong, M. Annie Saltarikos, David Somers, Yurong Chen

**Affiliations:** ^1^ Plant Biotechnology, Bayer Crop Science, W. St. Louis, MO, United States; ^2^ Agracetus Campus, Monsanto Company, Middleton, WI, United States; ^3^ Mystic Research, Monsanto Company, Mystic, CT, United States

**Keywords:** Zea mays, maize transformation, seed embryo explants (SEEs), meristem, *Agrobacterium*, organogenesis, genotype-flexible

## Abstract

A novel, efficient maize genetic transformation system was developed using *Agrobacterium*-mediated transformation of embryo explants from mature seeds. Seeds from field grown plants were sterilized and crushed to isolate embryo explants consisting of the coleoptile, leaf primordia, and shoot apical meristem which were then purified from the ground seed bulk preparation. The infection of relevant tissues of seed embryo explants (SEEs) by *Agrobacterium* was improved by the centrifugation of the explants. Transgenic plants were obtained by multiple bud induction on high cytokinin media, followed by plant regeneration on hormone-free medium. Three different selectable markers (*cp4 epsps, aadA*, and *nptII*) were successfully used for producing transgenic plants. Stable integration of transgenes in the maize genome was demonstrated by molecular analyses and germline transmission of the inserted transgenes to the next generation was confirmed by pollen segregation and progeny analysis. Phenotypic evidence for chimeric transgenic tissue was frequently observed in initial experiments but was significantly reduced by including a second bud induction step with optimized cytokinin concentration. Additional improvements, including culturing explants at an elevated temperature during bud induction led to the development of a revolutionary system for efficient transgenic plant production and genome editing. To our knowledge, this is the first report of successful transgenic plant regeneration through *Agrobacterium*-mediated transformation of maize mature SEEs. This system starts with mature seed that can be produced in large volumes and the SEEs explants are storable. It has significant advantages in terms of scalability and flexibility over methods that rely on immature explants.

## Introduction

Immature embryos have been the explants of choice for maize transformation since the first reports of regenerating fertile transgenic plants (Fromm et al., 1990; Gordon-Kamm et al., 1990; Walters et al., 1992; Songstad et al., 1996; Ishida et al., 1996). However, the utility of this system relies on the availability of constant, consistent source of high-quality donor plants for transformation. More importantly, a transformation system based on immature embryos is generally genotype dependent (Altpeter et al., 2016; Kausch et al., 2021). Physiological conditions of immature embryos, the size and stage of immature embryos, and environmental conditions that influence the physiological status of immature embryos, such as ear source, season, and pest infestation, contribute greatly to the variation in transformation frequency. Furthermore, isolation of immature embryos is typically a manual process that is slow and cumbersome. Initiation of experiments is dependent on the successful pollination of donor ears and requires approximately a two-month waiting period for donor plants to grow, and once generated, the explant material has a short transformability window and must be utilized within days.

Mature seeds offer an opportunity for a more efficient and reliable supply of explant material. Large amounts of mature seeds can be produced inexpensively in the field and stored under controlled environmental conditions, providing a constantly available and reliable supply of quality source material. Callus induction and plant regeneration from mature maize seed has been previously reported (Wang, 1987; Huang and Wei, 2004). In these reports, embryos were manually isolated from imbibed seeds and the plumule sections sliced longitudinally before starting tissue culture steps. Shoot regeneration frequency was improved in later studies when imbibed whole seeds were sliced longitudinally (the “split seed” method). Three different tissues, the scutellum, the coleoptilar ring and the shoot apical meristem in the split seed, were simultaneously exposed to culture medium (Al-Abed et al., 2006). This regeneration system has been implemented to produce transgenic plants (Sairam et al., 2003). In another approach, apical meristems from germinating seedlings were dissected and excised shoot apical meristems from seedlings or plantlets were directly used for transformation or to induce multiple buds for transformation (Gould et al., 1991; Zhong et al., 1992a; Zhong et al., 1992b; Zhong et al., 1996; Li et al., 2002; Zhang et al., 2002; Sticklen and Oraby, 2005). Alternatively, shoot meristems from 3-4 days old germinating seeds without dissection were used for transformation (Sairam et al., 2003). Transformation of Type I callus, induced from nodal sections of 7 to 10-day old maize seedlings, with *Agrobacterium* led to regeneration of transgenic plants (Sidorov et al., 2006). More recently, successful transformation of mature seed-derived embryo axes slices in maize was possible *via* intermediate embryogenic callus through the over-expression of maize Baby boom (*Bbm*) and Wuschel2 (*Wus2*) genes at the early stage of transformation process, and removal of these plant morphogenic genes at a later stage to enable regeneration of transgenic plants (Lowe et al., 2016).

Successful direct transformation of mature seed embryo explants of dicot species was initially obtained in soybean and cotton through particle bombardment (McCabe et al., 1988; McCabe and Martinell, 1993). These methods involved low throughput handling of explants for particle bombardment and screening of plants for presence of a transgene. A breakthrough in transformation of dicots came from the development of *Agrobacterium*-mediated transformation protocols with selectable markers, such as *aadA* for antibiotic selection, or *cp4 epsps* for herbicide selection. Transgenic plants were obtained by organogenesis pathway directly from meristem region of isolated SEEs without callus induction (Martinell et al., 2002; Ye et al., 2008; Ye et al., 2011; Calabotta et al., 2013; Chen et al., 2014; Chen et al, 2020).

Progress in SEE transformation in dicot species inspired us to develop a similar SEE transformation protocol for monocot species such as maize. In this manuscript, we present a novel, *Agrobacterium*-mediated transformation of SEEs from mature seeds, which provides significant advantages of storability and high flexibility for transgenic plant production over immature embryo-based transformation systems.

## Materials and methods

### Plant materials and explant preparation

**Figure 1:**
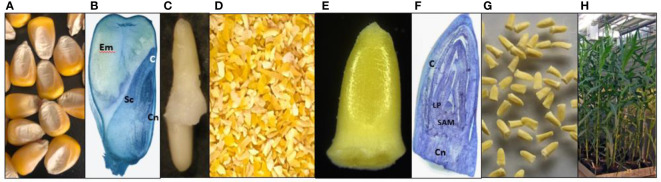
Maize seed embryo explants (SEE) for *Agrobacterium*-mediated direct transformation. **(A)** Maize dry seeds; **(B)** Longitudinal section of a maize dry seed cutting through embryo. Endosperm (Em), scutellum (Sc), coleoptile **(C)**, and coleoptile node (Cn) are indicated; **(C)** Manually isolated intact embryos with radicle and coleorhiza from an imbibed maize seed; **(D)** Crushed maize dry seed particles; **(E)** A magnified maize SEE; **(F)** Longitudinal section of an imbibed SEE with coleoptile **(C)**, leaf primordia (LP), shoot apical meristem (SAM) and coleoptile node (Cn); **(G)** Purified SEE for transformation; **(H)** healthy maize plants from SEE grown in green house. (Panels **B–F** were stained by 0.1% toluidine blue and made available by Mrs. Olivia Haragutchi).

Three inbred lines, LH244, BPL1, and BPL2, were used in this study to develop a transformation system using mature seed embryo explants. LH244 was recently released to the public for non-commercial use through USDA-GRIN. The remaining two lines, BPL1 and BPL2 are proprietary of Bayer. SEEs were produced through the grinding in GP-140 grinder of surface sterilized mature maize seeds (**Figure 1A**). In early experiments SEEs were manually picked with forceps following excision (**Figure 1B**). In later experiments, SEEs were sterilized and purified as follows: After excision dry SEEs were directly surface sterilized in 70% ethanol for 4 mins and rinsed with sterile water 4 times. Sterilized explants were soaked in autoclaved water and separated from the debris through flotation in water in a plastic container or a glass beaker (**Supplemental Figure S2**). Purified explants with or without prior treatment of 2mM potassium hydroxide (KOH) were rehydrated in the 1595 medium for 0.5 to 2 hours before inoculation with *Agrobacterium*. The rehydration medium 1595 was supplemented with 50 mg/L Nystatin and 10 mg/L thiabendazole (TBZ) to control potential fungal contamination.

### *Agrobacterium* strain and binary vectors

*Agrobacterium tumefaciens* nopaline strain AB32 was used for all experiments. The AB32 strain has VirG^N54D^ mutation which renders VirG constitutively active, removing the requirement for acetosyringone induction for monocot transformation (Ye et al., 2016). Binary vectors were electroporated into the AB32 competent cells followed by selection on LB medium with 30 mg/L gentamicin and 75 mg/L spectinomycin. The overnight culture was spun down when OD_600_ was around 1.0 followed by resuspending the *Agrobacterium* pellet in medium 1595 at an OD_600_ value between 0.3 to 0.6 for inoculation (Ye et al., 2011; Chen et al., 2014).

Maps and relevant element information for all plasmids is contained in **Supplemental Figure S1**. Information for pMON42073 containing *gus*A, *nptII*, *gfp* and *cp4 epsps* expression cassette was previously disclosed in detail (Armstrong et al., 2010). Plasmid pMON97367 is similar to pMON92726 (Ye et al., 2011) with additional 333 bp CaMV enhancer sequence inside the rice act1 promoter. Plasmid pMON138203 was made by adding the *CTP-aadA* expression cassette driven by enhanced CaMV 35S promoter and a transcriptional terminator sequence from the *Agrobacterium nos* gene into pMON97367 (Ye et al., 2008; Martinell et al., 2013). The binary vectors have RK2 *oriV* backbone with spectinomycin selection (Ye et al., 2011).

### Inoculation and co-culture

All media used in this work is listed in **Table 1**. For inoculation, mechanically or floatation-purified and rehydrated SEEs were submerged in 50 ml *Agrobacterium* inoculum in a Plantcon (MP Biomedicals, LLC). In some experiments, manually picked SEEs or floatation-purified SEEs were transferred into 2 mM KOH water solution in a beaker, stirred for 1 hour on a magnetic stirrer and neutralized by replacing with medium 1595 three times before *Agrobacterium* inoculation. The explants were then subjected to sonication in Honda W-113 ultrasonic Multi-Cleaner at 45 kHz for 2 min, followed by vacuum infiltration at 25 InHg for 5 min. The explants were transferred from Plantcon to 50 ml Falcon tube or 500 ml conical bottle and centrifuged at 291 g for 30 mins. After inoculation, the *Agrobacterium* suspension was removed completely, and explants were transferred to a piece of Whatman # 1 filter paper (85 mm) pre-moistened with 1.25-1.50 ml of co-culture media in a 25 x 100 mm Petri dish. In some instances, the co-culture media were supplemented with 0.005% Silwet L77, 50 µM lipoic acid or 50 mg/L Nystatin and 10 mg/L thiabendazole (TBZ). Co-culture media used in various experiments and treatments included media 1273, 1484 and 1595 supplemented with different types and levels of plant growth regulators, such as 4 mg/L 6-benzylaminopurine (BAP) and 2.2 mg/L picloram, 2-8 mg/L 2,4-dichlorophenoxyacetic acid (2,4-D), 10 mg/L indole acetic acid (IAA), or 10 mg/L naphthaleneacetic acid (NAA). The explants were co-cultured in Percival incubators at 23°C, relative humidity of 70%, light density of 90 µmol/m^2^s, and a photoperiod of 16-light and 8-h dark for 3-6 days. After co-culture, samples of explants were taken for GUS transient expression assay or GFP expression observation using a microscope.

**Table 1:** Media used for transformation of maize mature seed embryo explants.

Medium name	Base media	Cytokinin	Auxin	Other additives
1595	2/5 B5 macro salts + 1/10 B5 micro salts and vitamins	0	0	30 g/L dextrose + 3.9 g/L MES
1484	MS medium	1 mg/L BAP	2.2 mg/L Picloram	40 g/l maltose + 0.5 g/l casein hydrolysate + 1.95 g/l MES + 100 mg/l ascorbic acid;
1273	MS medium	3 mg/L BAP	10 mg/L Picloram	40 g/l maltose + 0.5 g/l glutamine + 0.75 g/l magnesium chloride + 0.1 g/l casein hydrolysate + 1.95 g/l MES +100mg/l ascorbic acid
CMSI-18	MS basal salts + B5 vitamins	10 mg/L BAP	1 mg/L 2,4-D	30 g/l sucrose + 0.69 g/l l-proline + 1 g/l casein hydrolysate + 2 mg/l glycine + 1 g/l MES + 400 mg/l carbenicillin + 200 mg/l cefotaxime + 100 mg/l timentin + *3.5 g/l agarose*
CMSI-43	MS basal salts + B5 vitamins	2 mg/L BAP	0.5 mg/L 2,4-D	30 g/l sucrose + 0.69 g/l l-proline + 1 g/l casein hydrolysate + 2 mg/l glycine + 1 g/l MES + 400 mg/l carbenicillin + 200 mg/l cefotaxime + 100 mg/l timentin +*3.5 g/l agarose*
2913	MS medium without nitrogen	0	0.5 mg/L 2,4-D + 2.2 mg/L Picloram	30 g/l sucrose + 1.64 g/l potassium sulfate + 1.65 g/l ammonium nitrate + 0.5 mg/l thiamine HCl + 1.38 g/l proline + 0.5 g/l casamino acid + 3.4 mg/l silver nitrate + 25 µM glyphosate 400 mg/l carbenicillin + 200 mg/l cefotaxime + 100 mg/l timentin + 3.5 g/l agarose
4141	MS basal salts + B5 vitamins	3 mg/L TDZ	2 mg/L Picloram	60 g/l sucrose + 0.5 g/l glutamine + 1.25 mg/l cuso4 ++ 30 µM glyphosate + 200 mg/l carbenicillin + 250 mg/l cefotaxime + + 100 mg/l timentin + 3.5 g/l agarose
1083	MS Medium	0	0	10 g/l glucose + 20 g/l maltose + 0.15 g/l + 0.15 g/l asparagine monohydrate + 0.1 g/l myo-inositol + 20 µM glyphosate, 400 mg/l carbenicillin + 200 mg/l cefotaxime + 100 mg/l timentin + 3.5 g/l agarose
4809	Woody Plant Medium	0	0	30 g/l sucrose + 0.69 g/l proline + 1 g/l MES + + 20 µM glyphosate, 400 mg/l carbenicillin + 200 mg/l cefotaxime + 100 mg/l timentin + 3.5 g/l agarose
3763	MS basal salts + B5 vitamins	0	0	30 g/l sucrose + 0.69 g/l proline + 2 mg/l glycine + 1 g/l MES + + 20 µM glyphosate, 400 mg/l carbenicillin + 200 mg/l cefotaxime + 100 mg/l timentin + 3.5 g/l agarose

### Induction and proliferation of multiple buds and regeneration of transgenic plants

After co-culture, the explants from each co-culture plate were transferred to five plates of the initial multiple induction media such as CMSI-18 with BAP 10 mg/L and 2,4-D 1 mg/L with or without selection and cultured at 28°C or specific condition outlined in a particular experiment, a light density of 60 µmol/m^2^s and a photoperiod of 16-light and 8-h dark. After initial bud induction for 7-14 days, growing explants from each plate were subsequently transferred to the same bud induction medium or different bud proliferation medium for 7-14 days before the transfer to various regeneration media for shoot regeneration and root differentiation. In some instances, growing explants from the initial bud induction medium were directly transferred to shoot regeneration medium without the bud proliferation medium. Bud induction medium and induction duration, and regeneration medium are described in individual experiments. Selection reagent, e.g., 75 mg/L paromomycin, or 100 mg/L streptomycin, or 25 µM glyphosate was added to the selection media during the bud proliferation step or plant regeneration for constructs containing *nptII*, *aadA* and *cp4 epsps* selectable markers respectively. Further details of the media are given in **Table 1**.

### Growth and development of transgenic plants in the greenhouse

Leaf tissue of the regenerated plants were assayed for GUS expression (Jefferson, 1989). Transgenic plants were initially transplanted to a small soil pot and subsequently to a large pot for further development in the greenhouse. Leaf tissue was collected for molecular analysis.

### Histochemical assay of GUS expression and GFP fluorescence microscopy

GUS assay for transient and stable transformation was conducted following published protocol (Jefferson, 1989). SEEs after co-culture were sampled for transient assay. Transgenic events were identified by assaying leaf tissue from regenerated plants for GUS expression. The GUS assay reactions were conducted at 37°C for a few hours or overnight. For observation of GFP expression, co-cultured explants were examined under a Leica microscope. For the detailed localization of GFP expression, co-cultured explants expressing GFP were selected under the fluorescence stereomicroscope. Explants were then placed in room temperature Tissue-Tek^®^ O.C.T. Compound (OCT), oriented for slicing, and then placed in the cryostat chamber to allow freezing. Once at -10 to -12°C, sections (40-70µm) were collected directly onto microscope slides and kept frozen. Frozen slides were placed directly into a Coplin jar containing 1x PBS with or without MgCl2 for about 5 mins. Excess OCT was wiped off as much as possible using a Kimwipe before adding VectaShield mounting media to the sections and placing the cover slip. Sections were analyzed and imaged using confocal microscopy and image capture software (laser intensity set at 15-20%, gain set at 6.9).

To determine the frequency of germline events, pollen grains from flowering plants were stained for GUS expression. The presence of GUS positive pollen indicates germline transformation. Transgenic plants were either selfed or crossed to wild type inbreds. Seedlings derived from T1 or F1 seeds were tested for GUS expression.

### Southern blot and progeny analyses

For Southern blot, the leaf samples from greenhouse growing plants were collected and extracted as previously described (Ye et al., 2011). Southern blots were carried out with DIG-labeled probe according to the manufacturer instruction (Roche, Penzberg, Germany) as previously described (Dellaporta et al., 1983). pMON42073 was used as a template for DIG probe labeling with PCR using DIG synthesis kit from Roche (#13015220) and *PfuUltra II* fusion high fidelity DNA polymerase (Agilent, Santa Clara, USA) according to the manufacturer instructions. The following primer pairs were used for probe synthesis: 5’ ACGATATCACCGTGGTGACGC 3’ (*gusA* forward) and 5’ cactccacatgtcggtgtaca 3’ (*gusA* reverse) amplify a 1014 bp *gusA* fragment; 5’ tcgcatgattgaacaagatgga 3’ (*nptII* 5’ forward) and 5’ agagtcccgctcagaagaactc 3’ (*nptII* 3’ reverse) amplify a 808 bp *nptII* fragment; 5’ cattcccggcgacaagtcgatc 3’ (*cp4 epsps* forward) and 5’ tcgtatcggagagttcgatcttcg 3’ (*cp4 epsps* 3’ reverse) amplify a 1288bp *cp4 epsps* fragment. The labeled PCR products were fractionated in 1% agarose gel, and the specific probe bands were purified using Qiagen spin columns (Qiagen, Germantown, MD, USA).

To estimate the copy number of T-DNA insertions in the transgenic maize events, the *cp4 epsps* and *gusA* assays were conducted by Taqman^®^ quantitative analysis using a probe specific to the *cp4 epsps* or *gusA* gene according to the manufacturer’s instruction (Applied Biosystems, Foster City, CA). The Taqman^®^ probe was designed and validated by the manufacturers. The Taqman^®^ detection probes of the *gusA* were described previously (Vaghchhipawala et al., 2018). For *cp4 epsps* selectable marker copy number determination, the primers 5’-ACGATTTCGACAGCACCTTCA-3’ (forward) and 5’- GTCACCGTCTTCCGATTTCAC-3’ (reverse) amplifying the *EPSPS-CP4* gene, and further detected by minor grove binding Taqman^®^ probe 6FAM-ACGCCTCGCTCACAAAGCGCC, were used. The single copy transgenic events were counted when both *gusA* and *cp4 epsps* Taqman^®^ detection were concordant as single copy.

### Statistical analysis

For protocol development, experiments were conducted as randomized complete block designs with three or four replicates, data were transformed by arcsin square root and analyzed using SAS program (Proc Anova, SAS/STAT User’s Guide, 1989) where appropriate. Means were tested using protected Student t-test and considered statistically significant from each other when P was <0.05.

## Results

### Maize seed excised embryo structure

Dry maize seeds consist of starchy endosperm and an embryo buried in the mature scutellum tissue (**Figures 1A, B**). The detailed anatomy annotations for maize embryo structure have been described (Kiesselbach, 1949). The intact maize embryo with both plumule (leaf primordia and shoot apical meristem) and radicle (**Figure 1C**) is difficult to excise manually or mechanically from a dry seed. The shoot apical meristem (SAM) is wrapped within multiple layers of leaf primordia and the coleoptile, as shown in the longitudinal section across the meristem (**Figures 1B, F**). We used a grinder to crush dry maize seeds into small pieces (**Figure 1D**). Since the middle part of the maize seed, consisting of the first internode, lateral seminal root and scutellar node are tightly attached to scutellum tissue, only the upper part of dry embryo, consisting of the coleoptile, the plumule (leaf primordia and apical meristem), and part of the coleoptile node, was isolated intact, approximately 1x2 mm (width by length, **Figure 1E**). They can be purified from other non-embryo materials by manually picking with a pair of forceps (**Figure 1G**). Alternatively, explants can be purified at large scale using floatation methods (**Supplemental Figure S2**). Since the radicle, root apical meristem, root cap, and coleorhiza were lost during processing, we tested germination and viability by plating the purified SEEs in a PlantCon™ containing 1083 medium. We observed germination rates up to 90%, depending on the batch. Healthy maize plants were obtained and transplanted into a greenhouse to demonstrate normal development and fertility (**Figure 1H**), which indicates the presence of intact meristems.

### Effective *Agrobacterium* infection in meristematic cells of SEEs

**Figure 2:**
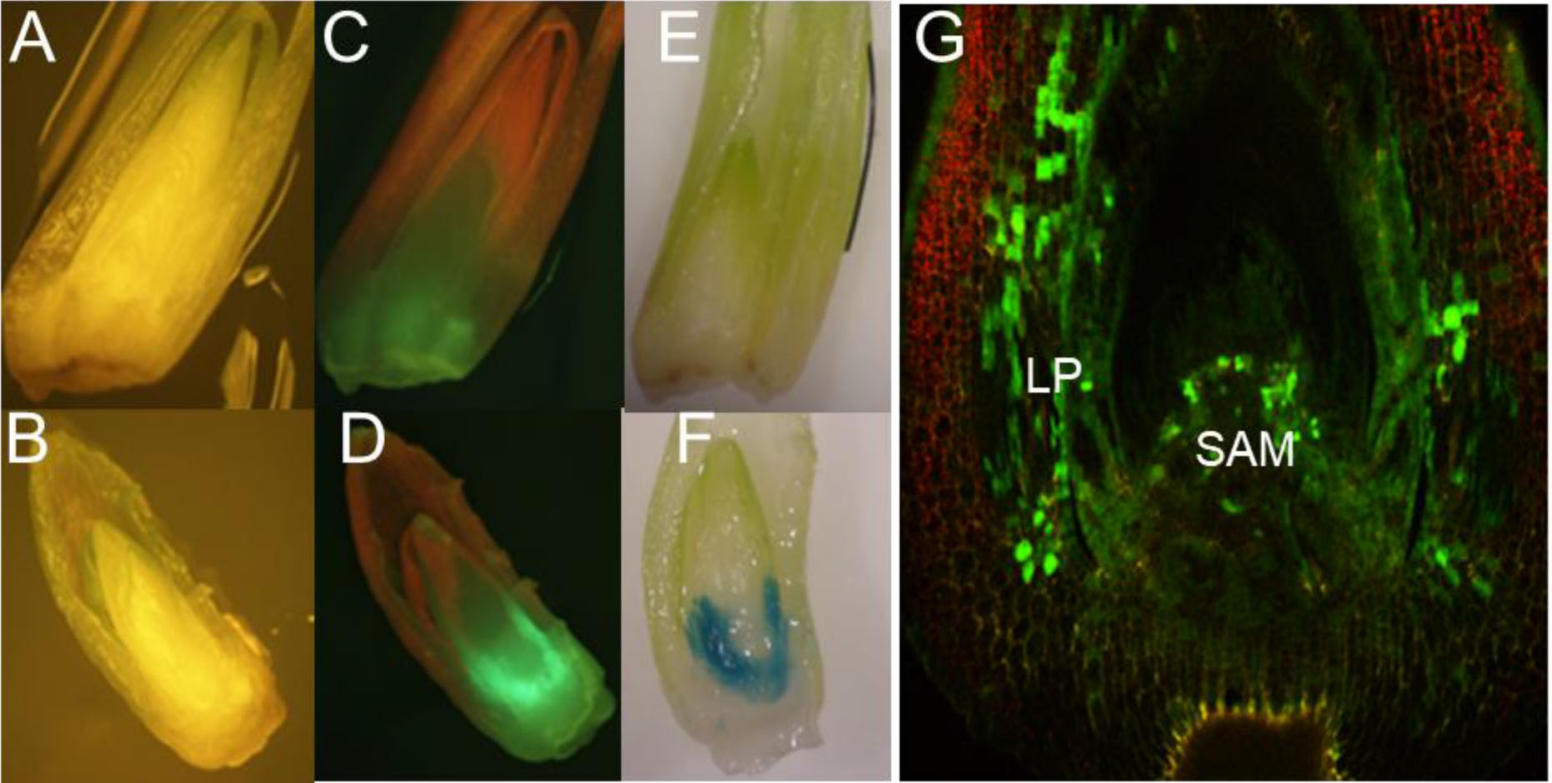
T-DNA delivery into relevant tissues of maize SEEs by centrifugation during inoculation. **(A)** *Agrobacterium* inoculation by centrifugation at 1 x g with visual light control for GFP; **(B)** *Agrobacterium* inoculation by centrifugation at 291 x g; **(C)** GFP expression after *Agrobacterium* inoculation by centrifugation at 1 x g; **(D)** GFP expression after *Agrobacterium* inoculation by centrifugation at 291 x g; **(E)** GUS expression after *Agrobacterium* inoculation by centrifugation at 1 x g; **(F)** GUS expression after *Agrobacterium* inoculation by centrifugation 291 x g; **(G)** Detailed view of GFP expression of a SEE inoculated by centrifugation in the presence of *Agrobacterium*, under a confocal microscope, LP-leaf primordia, SAM-shoot apical meristem. pMON42073 (**Supplemental Figure S1**) was used for these experiments.

Efficient T-DNA delivery by *Agrobacterium* into cells of regenerable tissue is a key step in the development of transformation protocols for any plant species. In the case of maize SEEs, the shoot apical meristem (SAM) region of the embryo is covered by the coleoptile and multiple layers of leaf primordia. To this end, various physical and chemical approaches to improve the accessibility of *Agrobacterium* to meristematic cells were tested. Those approaches included vertical slicing of rehydrated SEEs, pretreatment of rehydrated SEEs with Silwet L77, DMSO or KOH, sonication and/or vacuum infiltration during *Agrobacterium* inoculation. Histochemical GUS (β-glucuronidase) assay and GFP (green fluorescence protein) observations were conducted following the co-culture. Among these inoculation treatments, pretreatment of SEEs with 2 mM KOH solution for 1 hour with gentle stirring on a magnetic stirrer followed by sonication during *Agrobacterium* inoculation dramatically improved transient expression in coleoptile, as compared to the control (**Supplemental Figure S3**). Based on these preliminary results, we decided to investigate other methods to improve the *Agrobacterium* infection in maize SEEs. To our surprise, the most significant improvement of *Agrobacterium* transient delivery of the T-DNA came from the centrifugation of SEEs during *Agrobacterium* inoculation. SEEs were bisected after co-culture with *Agrobacterium* and observed under a dissecting microscope to determine if effective *Agrobacterium* infection was in the meristematic cells of SEEs. As shown in **Figures 2A–E**, the control without centrifugation had little to no *gfp* or *gusA* expression. In contrast, transformation of SEEs subjected to centrifugation with *Agrobacterium* cultures showed both strong *gfp* and *gusA* expression in the leaf base area and meristem region (**Figures 2D, F**). Detailed confocal microscopy studies further indicated that *gfp* expression was most prevalent in leaf base and shoot apical meristem (SAM) region (**Figure 2G**). We further explored the impacts of centrifugation, sonication, and temperature on T-DNA delivery of the *gusA* gene during *Agrobacterium* inoculation (**Supplemental Figure S4**) using MUG fluorometric assay for quantitative analysis of beta-glucuronidase (GUS) activity three days after the various treatments. A combination of centrifugation at 291 g and sonication (1 minute at 23 °C) was found to be most significant contributor to functional T-DNA delivery. Effective *Agrobacterium* infection to the relevant tissue of meristem explant through centrifugation was also demonstrated in multiple genotypes (**Supplemental Figure S5**). Manipulation of co-culture medium and co-culture condition further improved *Agrobacterium* infection. For example, B5-based co-culture medium (1595) consistently improved *Agrobacterium* infection as compared to MS-based co-culture medium (1484), which is routinely used for maize immature embryo transformation (**Supplemental Figure S6**).

### Multiple bud induction and regeneration of the first transgenic plants from SEEs

**Figure 3:**
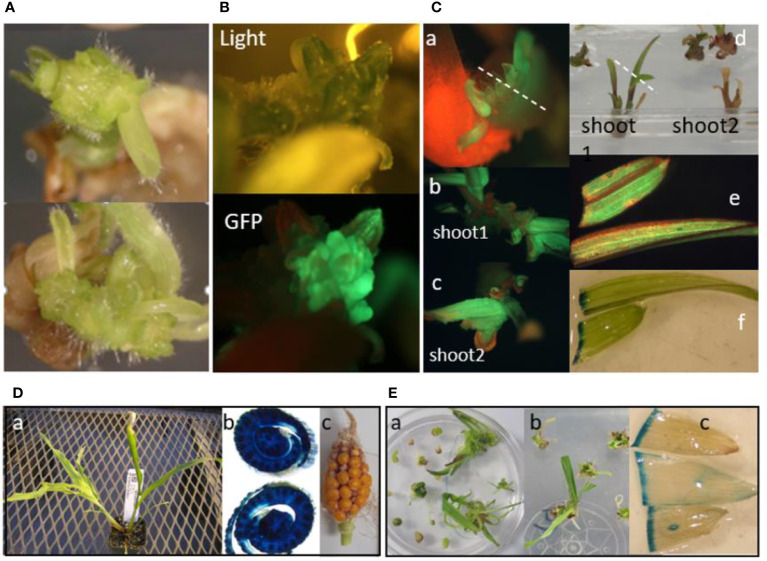
Multiple bud induction and direct transformation of maize SEEs for transgenic plant regeneration on high cytokinin medium CMSI-18. **(A)** Multiple bud formation from 2 individual SEEs after 3 weeks on CMSI-18 without selection; **(B)** A bud expressing GFP was selected on CMSI-18 with 75 mg/L paromomycin for 60 days after co-culture, whole clump is about 1 cm in diameter; **(C)** GFP-expressing multiple buds from maize SEEs direct transformation using streptomycin selection in CMSI-18. a: Multiple bud formation after 5 weeks on streptomycin selection medium, dot line indicates cutting into 2 buds for elongation of shoot 1 b: and shoot 2 (c), d: Two GFP positive shoots in rooting medium, dotted line indicates samples for GFP observation (e) and GUS staining (f); **(D)** A transgenic shoot from paromomycin selection in CMSI-18. a: the shoot in soil, b: GUS staining of transverse section of the old stem, c: ear recovered from feminized tassel of transgenic shoot in a; **(E)** A glyphosate resistant shoot from direct transformation of SEEs. a: 7 weeks after inoculation and selected on hormone-free medium 1083 with 20 mM glyphosate, b: the rooting shoot on the same media, c: GUS-stained leaves from the shoots in soil.

Having demonstrated that *Agrobacterium* can directly deliver T-DNAs into the internal meristematic region of the embryo covered by coleoptiles, we next focused on developing a system for stable transformation of cells and regeneration of transgenic shoots *via* the organogenesis pathway, specifically the induction of transgenic buds that can be elongated into shoots. Numerous exploratory experiments were performed with various combinations of cytokinin and auxin concentrations and duration of exposure to the hormones to identify conditions that would result in the production of stably transformed bud structures from meristematic tissues. In one experiment, approximately 600 hand-picked maize SEEs were pretreated with 2 mM KOH followed by inoculation with *Agrobacterium* AB32/pMON42073 suspension in medium 1595 containing 5 mg/L 2,4-D. Following co-culture, the explants were transferred onto CMSI-18 medium (10 mg/L BAP and 1 mg/L 2,4-D) containing 75 mg/L paromomycin selection for bud induction, sub-cultured on the same medium every 3 weeks and checked for *gfp* expression periodically. Multiple bud structures were observed after one or two weeks on the bud induction medium (**Figure 3A**). Approximately after 60 days on the CMSI-18 medium, one green bud clump, which was around 1 cm in diameter, expressed GFP (**Figure 3B**). This was the first indicator that meristematic tissue inside the SEE was able to be transformed by *Agrobacterium* and developed into a multiple bud/shoot structure. Unfortunately, this clump could not be regenerated.

After demonstrating that the maize SEE was directly transformable to form GFP positive buds, we then initiated similar experiments with *Agrobacterium* strain AB32/pMON138203 containing the *aadA* selectable marker for streptomycin selection. Approximately 600 hand-picked SEEs were sterilized with 70% ethanol for 4 min, pretreated with 2 mM KOH, mixed with *Agrobacterium*, vacuumed once at 15 InHg for 1 min, sonicated for 1 min, and subjected to centrifugation at 291 g for 30 min. After co-culture, green viable explants were transferred onto CMSI-18 containing 100 mg/L streptomycin for selection. Multiple buds observed after five weeks of selection on a medium containing 100 mg/L streptomycin (**Figure 3A**) expressed GFP (**Figure 3B**). For elongation of shoots, GFP positive buds were cultured on hormone-free medium 1083. Four weeks later, GFP positive shoots were formed (**Figures 3C–b, c**). Expression of *gfp* and *gusA* genes was observed in leaves of two developing shoots (**Figures 3C–d, e, f**). Unfortunately, the shoots did not produce roots and died after 2 months. The first evidence for regenerating stably transformed plants came from an experiment using the *Agrobacterium* strain AB32/pMON42073 containing the *nptII* and *epsps-cp4* selectable markers. Approximately 20,000 SEEs were purified by floatation and transformed with the *Agrobacterium* suspension. One plantlet emerged after SEEs were cultured on bud induction medium CMSI-18 for two weeks and sub-cultured on CMSI-43 medium containing 75 mg/L paromomycin selection for six weeks. Root formation occurred after the plantlet was transferred to medium 1083 containing 20 µM glyphosate (**Figures 3D–a**). Testing for expression of the *gusA* gene showed uniform expression (**Figures 3D–b**). This plant was able to set seed, albeit through a tassel seed phenotype (**Figures 3D–c**). In another experiment, about 20,000 SEEs were inoculated by centrifugation in the presence of the *Agrobacterium* suspension of AB32/pMON97367 containing the *epsps-cp4* marker and co-cultured for 3 days on medium 1595 with 5 mg/L 2,4-D. Three shoots were obtained by bud induction on CMSI-18 with 30 mM glyphosate selection for 6 weeks (**Figures 3E–a, b**). One of the plants was rooted in the hormone-free medium 1083 containing 20 mM glyphosate. Leaf samples were stained for verifying expression of the *gusA* gene (**Figures 3E, c**).

### Shorter multiple bud induction and lower cytokinin in media leads to reproducible transgenic plant production with normal fertility

**Figure 4:**
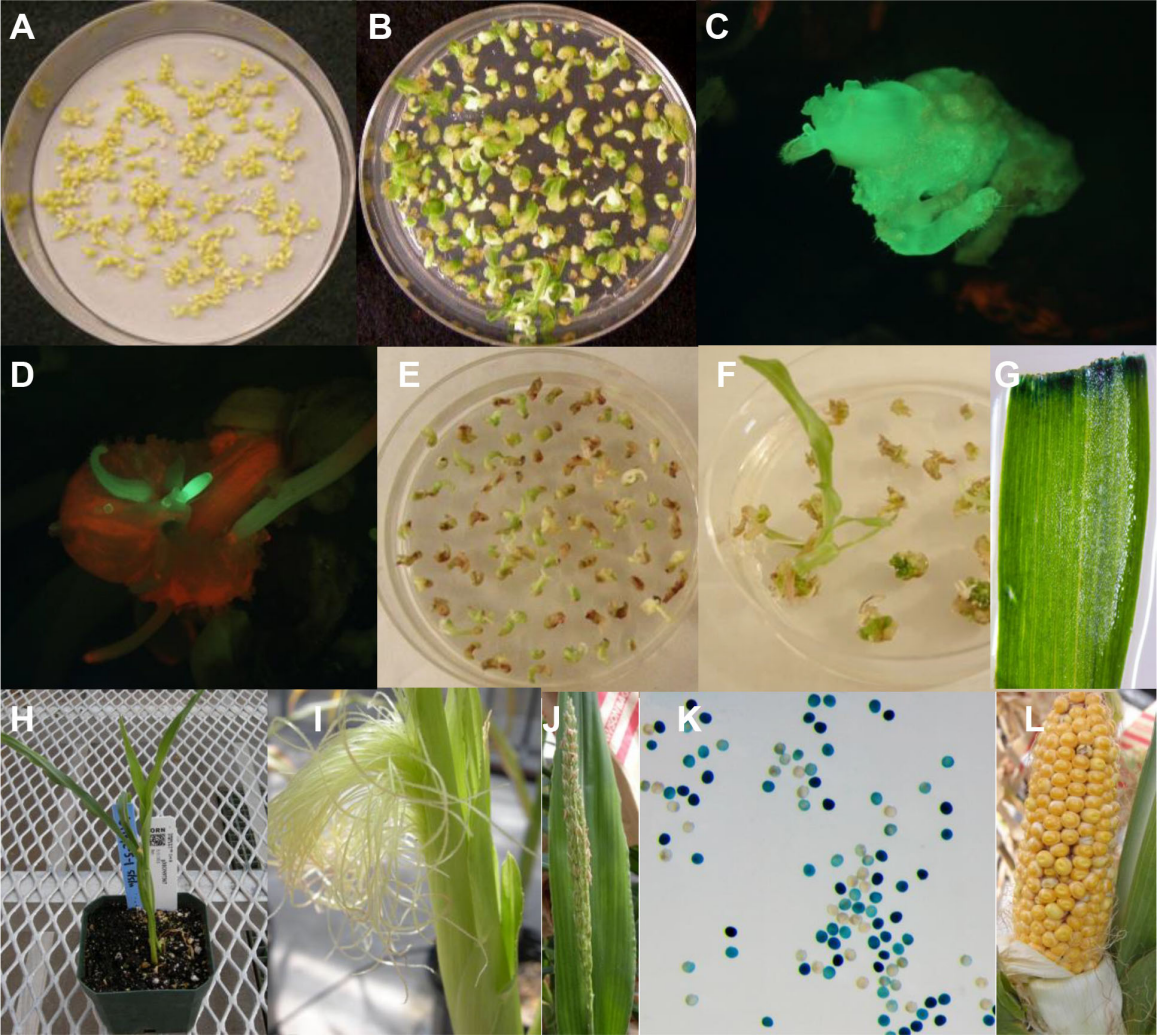
Regeneration of fertile events from multiple buds. **(A)** SEEs after co-culture for 3-6 days; **(B)** Bud formation from SEEs on bud induction medium 1484 containing 4.0 mg/L BAP and 2.2 mg/L picloram for two weeks; **(C)** A GFP positive bud formation from SEEs cultured on1484 medium containing 4.0 mg/L BAP and 2.2 mg/L picloram; **(D)** Elongating buds expressing GFP*;* **(E)** Buds induced on induction medium were transferred to bud proliferation medium 2913 containing 1.0 mg/L 2,4-D, 2.2mg/L picloram, and 25 µM glyphosate for 4 weeks; **(F)** Regeneration of transgenic plants 2-4 weeks after transfer of resistant buds to regeneration medium 1083 containing 25 µM glyphosate; **(G)** Leaf from regenerated plants expressing GUS; **(H)** Transgenic plants in soil pots in greenhouse; **(I)** Normal silk formation; **(J)** Normal tassel formation; **(K)** GUS assay of pollen to confirm germline transformation; **(L)** Normal seed set on transgenic event.

In a series of independent experiments with pMON42073, pMON138210, and pMON97367, we discovered that a shorter duration of bud induction on a lower cytokinin-containing medium followed by bud proliferation led to the development of a protocol reproducibly regenerating stable transgenic plants with normal fertility. After co-culture, explants grew and turned light green in color (**Figure 4A**) and were subsequently transferred onto bud induction medium containing a lower cytokinin/auxin level and ratio, i.e., 4 mg/L BAP and 2.2 mg/L picloram for two weeks where they appear visibly green (**Figure 4B**). Using fluorescence microscopy, we observed GFP positive multiple buds (**Figure 4C**) induced under this moderate cytokinin condition, which further proliferated and elongated in the bud induction medium (**Figure 4D**). Explants that were green in visible light were then transferred to a bud proliferation medium containing 1 mg/L 2.4-D and 2.2 mg/L picloram with 25 μM glyphosate, or 75 mg/L paromomycin or 100 mg/L streptomycin depending on the vector used in the experiment, for two-four weeks to encourage the development and proliferation of transgenic buds. The surviving explants with multiple buds (**Figure 4E**) were transferred to hormone free regeneration medium. Shoots were regenerated within two to four weeks of the transfer (**Figure 4F**) with transgenic plants easily identifiable by GUS assay of leaf tissue (**Figure 4G**). Plants with healthy root systems were transplanted to small soil pots (**Figure 4H**) for about 1 week for further root development and acclimatization before being transplanted to large pots where the plants entered the reproduction cycle. Silk (**Figure 4I**) and tassel formation (**Figure 4J**) on transgenic plants occurred approximately 6-7 weeks after transplanting with GUS assay of pollen (**Figure 4K**) used to determine germline transformation. Seeds could be harvested from pollinated ears in 5-6 weeks after pollination (**Figure 4L**). Under optimal conditions, the whole transformation process from initial inoculation to transplanting of plants to soil took about 10 weeks.

Three different selectable markers were used in these experiments to produce transgenic plants. Experiments used between 80,000 and 100,000 SEEs for each selectable marker, and produced 18, 26, or 102 transgenic plants for each selectable marker, with transformation frequencies ranging from 0.02% to 0.13% (**Table 2**).

**Table 2:** Regeneration of transgenic plants from three different selectable markers *cp4, aadA* and *nptI*I.

Selectable marker	Selective agent	# of seed embryo explants	# transgenic plants	% TF
cp4	25 µM glyphosate	80,000	102	0.128
aadA	100 mg/l streptomycin	100,000	26	0.026
nptII	75 mg/l paromomycin	90,000	18	0.020

### Molecular characterization of transgenic plants and progeny analyses

**Figure 5:**
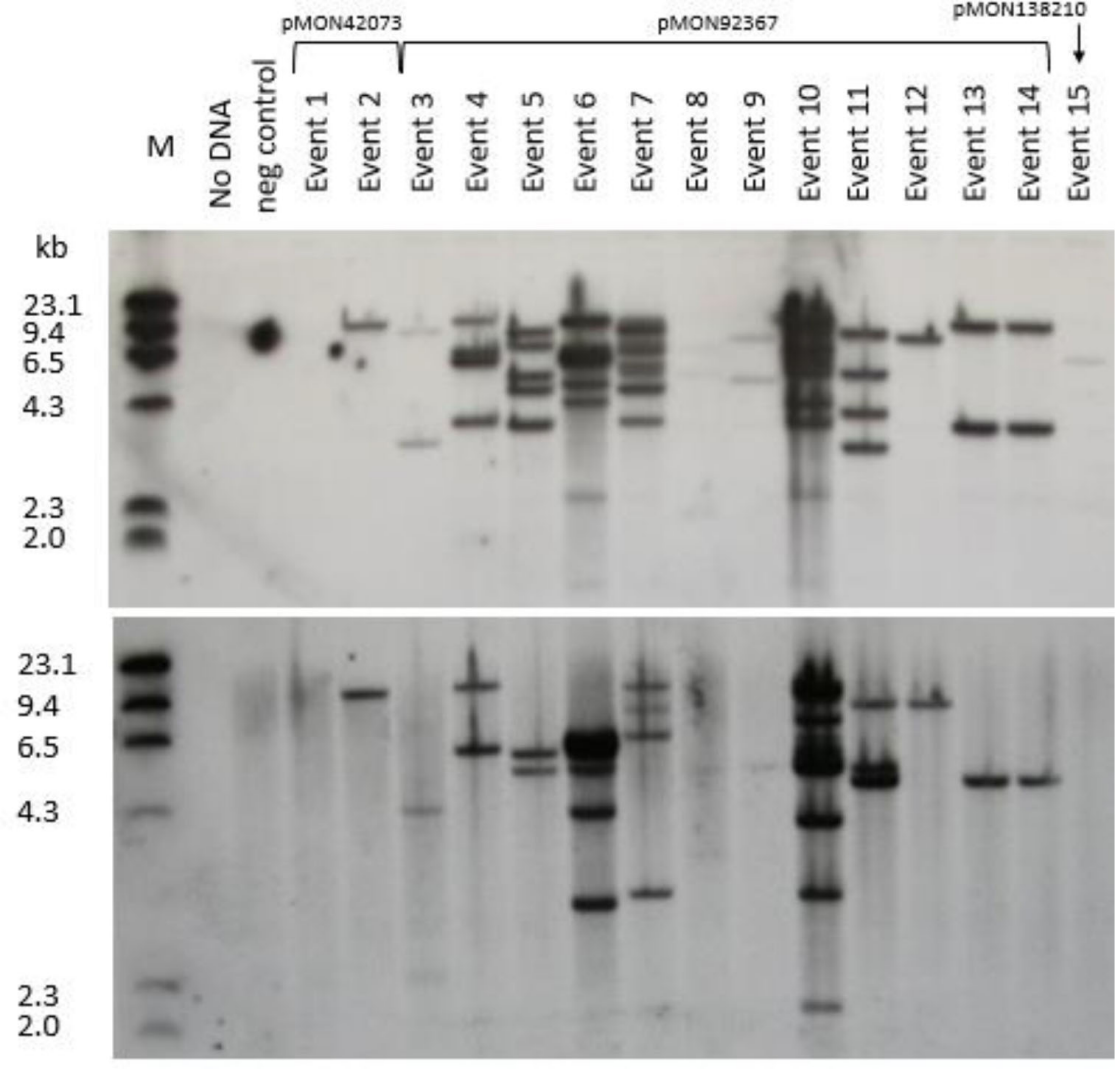
Southern blot analysis of 15 primary events from pMON42073, pMON92367, and pMON138210. Information about vector maps, *HindIII* restriction site location, and probe fragments are described in **Supplemental Figure S1**. The top panel was hybridized with a *gusA* probe and the bottom panel was hybridized with a *cp4 epsps* probe.

Southern analyses were conducted on 15 primary transformants (hereafter referred to as “events”) derived from transformation with three vectors containing three different selectable makers, *cp4 epsps*, *aadA* and *nptII* (**Figure 5**). The results of hybridization with *gusA* probe are shown in the top panel and with *cp4 epsps* probe presented in the bottom panel. Events 2, 12, and 15 appear to be single copy events while events 3, 9, 13 and 14 appear to have two copies of the *gusA* gene. All remaining events showed multiple hybridization bands except that event 1 was apparently a non-transgenic escape. Events 13 and 14 had similar banding patterns and were considered two subclones, probably representing two shoots derived from a single inoculated SEE. In summary, approximately 50% of events from this set of samples had 1-2 copies of *gusA* gene. Hybridization with *cp4 epsps* further confirmed the results for most events transformed by pMON42073 and pMON97367. However, it is interesting to note that the Southern banding pattern with the *cp4 epsps* probe was different from *gusA* probe for some events. This may indicate partial transfer of T-DNA or T-DNA re-arrangement. Nevertheless, the Southern analyses show that transgenic events from transformation of SEEs through a multiple bud pathway had stable T-DNA integration into the genome with multiple events appearing to have a single copy of the transgenes. GUS assay of pollen from six events showed that five of them i.e., events 5, 11, 12, 13, and 15 had a segregation pattern that was consistent with germline transmission of transgenes. Event 7 did not have positive GUS staining in pollen. That was most likely caused by transgene silencing due to the presence of >8 copies of the *gusA* gene. Several plants had very faint bands despite loading equal amounts of genomic DNA. We attribute this to the observation that some plants were genetically chimeric, with regard to the transgene insert (see below). Progeny analysis of wild type inbred by transgenic events further confirmed germline transmission of transgenes into the next generations. Eight out of 10 events transformed using *cp4 epsps* selection and all events transformed using *nptII* and *aadA* selection had normal Mendelian segregation pattern (**Supplemental Table S1**).

### Occurrence of chimeric transgenic plants in primary transformants

**Figure 6:**
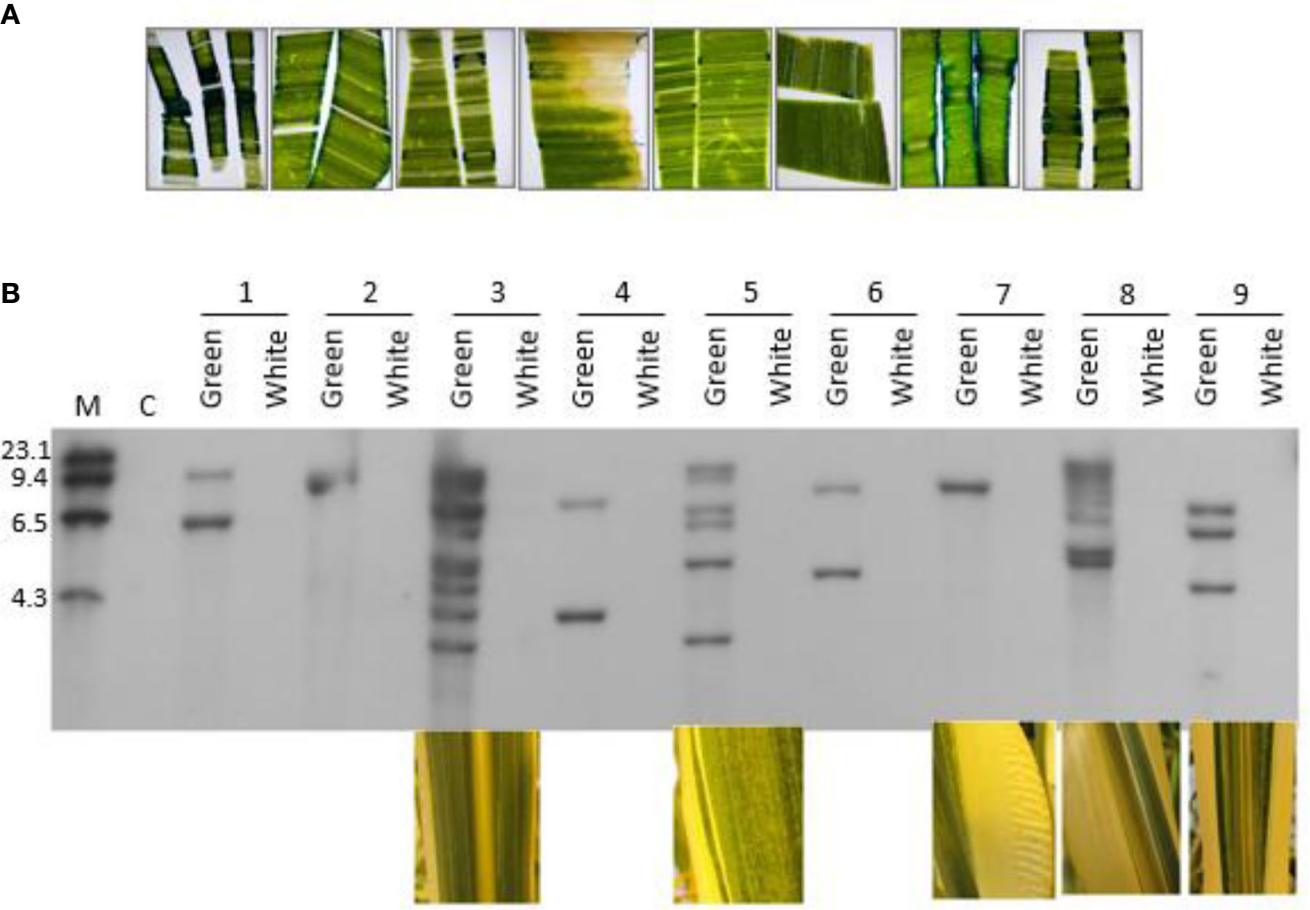
Phenotypic and molecular evidence for chimeras in regenerated plants from direct transformation of seed embryos; **(A)** Non-uniform expression of the *gus*A gene in leaf tissue from seven independent transgenic events, transformed with pMON97367; **(B)** Southern blot analysis of nine independent transgenic events. Green and white leaf sectors from individual events were dissected with a scalpel and separated prior to extraction of genomic DNA to test for the presence of transgenic insertions in each sector. Photos of the sectors that were used in the Southern blot experiment are shown below five of the events. M: Molecular marker size ladder. C: Negative control, a non-transgenic plant. Genomic DNA was digested with *HindIII* and probed with a fragment of the *gusA* gene. See **Supplemental Figure S1** for more information.

Phenotypes were observed in the primary transformants suggesting that the plants were comprised of chimerically transgenic tissue. First, we observed a chimeric phenotype based on non-uniform expression of the *gusA* gene in leaf tissue (**Figure 6A**). Typically, the sporadic, discontinuous blue staining along the cut edge of the leaf is indicative of chimerism when leaf tissues were stained with X-Gluc. We also observed it when using glyphosate as a selection agent. Plants regenerated from transformation with the *cp4 epsps* selectable marker gene that are chimerically transgenic can be identified phenotypically by the presence of white stripes due to glyphosate-associated bleaching of non-transgenic sectors. Chimeric events were identified during regeneration, at vegetative and reproductive development stages by a visible phenotype of white stripe (**Supplemental Figure S7**). As shown in **Figure 6B**, the correlation between transgene integrations and chimeric sectors was confirmed by Southern blots using DIG-labeled *cp4 epsps* marker probe. Individual leaves from nine individual phenotypically chimeric events were dissected to separate the white sectors from the green sectors. Genomic DNA was extracted separately from the sectors and used in a Southern blot analysis, probing for the presence or absence of a genetic element (*gusA*) of the T-DNA. The hybridization signal aligns well with the phenotype, confirming that the phenotypic chimerism is due to underlying genetic chimerism of the transformed tissues.

### Elevated temperature treatment at the bud induction improves transformation frequency

**Figure 7:**
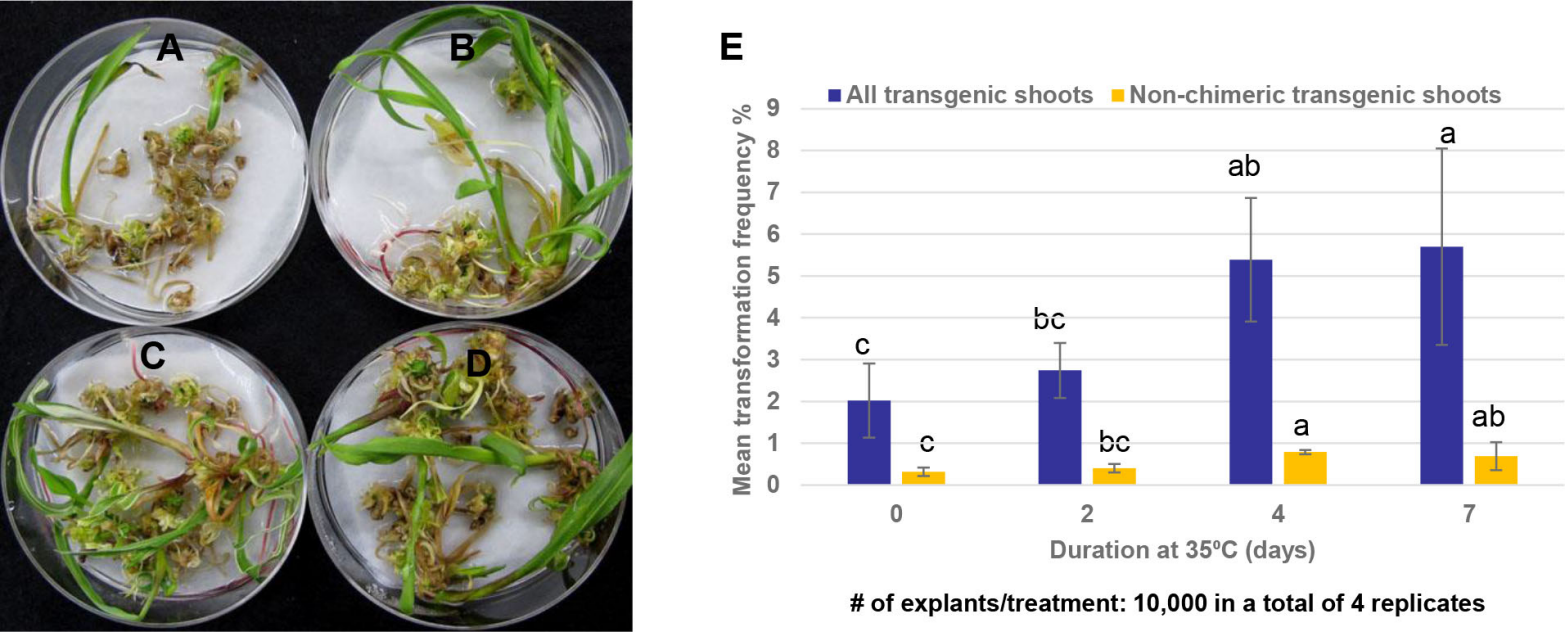
Effect of temperature on shoot bud formation. **(A)** Transgenic shoot regeneration when 1^st^ bud induction was conducted at 28°C for 7 days; **(B)** Transgenic shoot regeneration when SEEs were initially cultured at 35°C for 2 days before transfer to 28°C for an additional 5 days on 1^st^ bud induction; **(C)** Transgenic shoot regeneration when SEEs were initially cultured at 35°C for 4 days before transferring to 28°C for additional 3 days on 1^st^ bud induction **(D)** Transgenic shoot regeneration when SEEs were initially cultured at 35°C for 7 days on 1^st^ bud induction; **(E)** Culturing SEEs at 35°C for 4-7 days at the 1^st^ bud induction increased both the overall transformation frequency (TF) and the transformation frequency of non-chimeric plants.

Evidence from experiments summarized in **Figure 3** showed that long exposure of maize SEEs on the bud induction medium containing high cytokinin/auxin ratio e.g., CMSI-18 with 10 mg/L BAP and 1 mg/L 2,4-D led to high frequency of bud induction. However, bud induction on the high cytokinin media for more than 6 weeks was found to be associated with difficulty in shoot and root elongation (**Figure 3**) as well as undesirable phenotypes of the regenerated plants, e.g., tassel seed phenotype. Reduction of multiple bud induction time on CMSI-18 medium to two weeks at 28 °C followed by regeneration on hormone-free medium with 25 μM glyphosate selection led to direct rooting shoots in 4 weeks on the selection medium. We next tested the impact of temperature on bud induction and transformation frequency. In one experiment, SEEs were cultured on CMSI-18 for bud induction at 35°C for 2, 4 and 7 days after co-culture with *Agrobacterium*, and then transferred to 28°C for the remaining bud induction duration. For the control treatment, co-cultured SEEs were cultured at 28°C for two weeks bud induction (0 day at 35°C treatment). Results showed that culture of explants at 35°C for 4-7 days during the bud induction was sufficient to increase transgenic shoot regeneration as compared to culture of explants at 28°C continuously (**Figures 7A–D**). It was noted that culturing at an elevated temperature for 4-7 days during bud induction did not appear to have impact on chimera frequency. The chimera frequency in the regenerated transgenic events was in the range of 70-80% (**Figure 7E**).

### Reduction of the frequency of chimeric plants and improvement of overall transformation frequency through optimization of bud induction duration and medium composition

**Figure 8:**
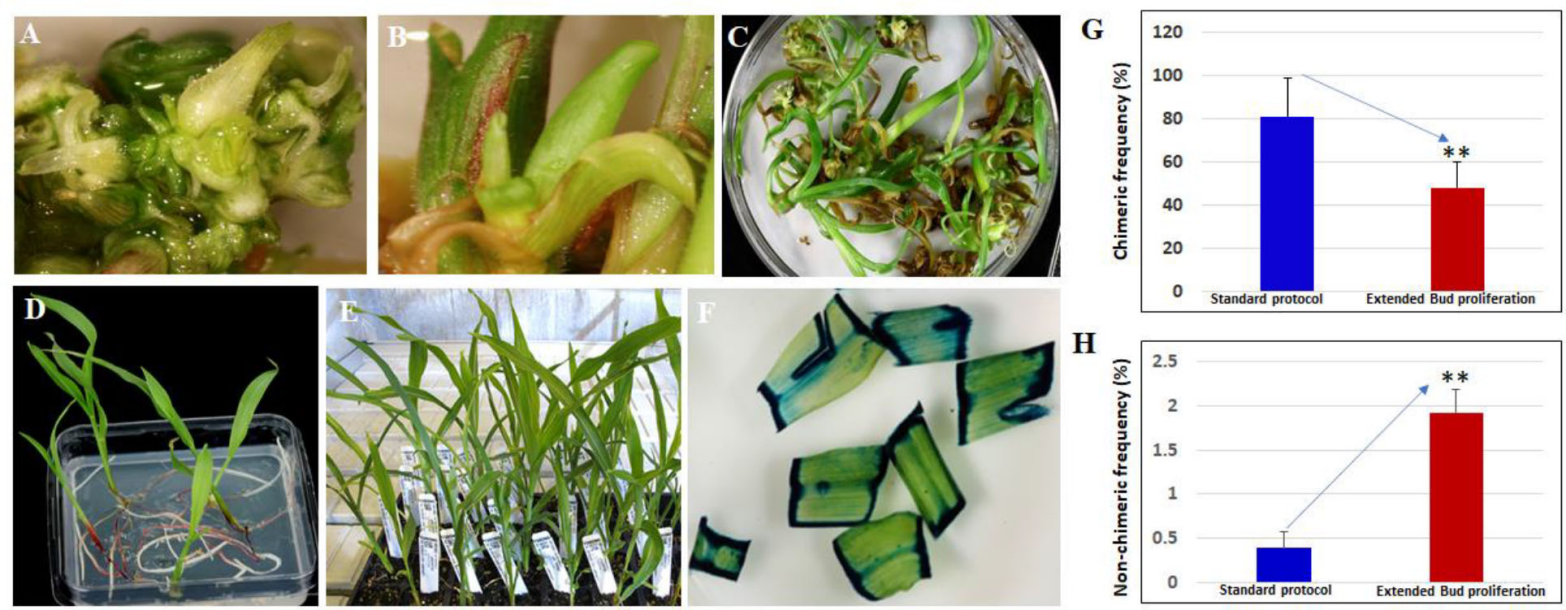
Reduction of chimeric event through the introduction of the extended bud proliferation step with selection and optimized hormone combination. **(A**, **B)** proliferation of glyphosate-resistant shoot buds on bud induction medium 4141 containing 3 mg/L thidiazuron, 2 mg/L picloram, and 30 mM glyphosate for two weeks; **(C)** Regeneration of glyphosate-resistant shoots on regeneration medium containing 20 mM glyphosate; **(D)** Glyphosate-resistant shoots developed a strong root system on rooting medium; **(E)** Transgenic plants with normal green leaf phenotype in plug; **(F)** *gusA* expression in non-chimeric glyphosate-resistant shoots; **(G)** Bud proliferation induction medium significantly (p<0.01) reduced the regeneration of chimeric shoots; **(H)** bud proliferation medium significantly (p<0.01) improved the overall production of non-chimeric events. ** indicates significance at <0.01 level.

The frequency of plants with a visible chimeric leaf phenotype was as high as 80% in the initial experiments. To reduce the frequency of chimerism of transgenic plants from SEE direct transformation and to promote the regeneration of non-chimeric events, the bud induction duration and medium composition were optimized. For the control treatment in this experiment, SEEs were cultured on bud induction medium CMSI-18 for two weeks without selection before being transferred to the regeneration medium in the presence of glyphosate. For the new protocol, buds induced on CMSI-18 medium for one or two weeks were transferred to 2nd bud induction medium 4141 containing 3 mg/L thidiazuron (TDZ), 2 mg/L picloram and 30 µM Glyphosate). Earlier glyphosate selection of multiple buds on the bud proliferation medium with the optimized cytokinin/auxin combination for two more weeks allowed the proliferation of green and healthy transgenic buds (**Figures 8A, B**), which converted into healthy green transgenic shoots on glyphosate-containing regeneration medium (**Figure 8C**). Glyphosate-resistant shoots with reduced phenotypic chimerism developed a strong root system after their transfer to the rooting medium (**Figure 8D**). Shoots developed into healthy and normal plants with lower chimerism in plugs (**Figure 8E**). GUS expression studies on hundreds of events produced *via* optimized 2^nd^ bud induction produced mostly non-chimeric plants lacking white sectors on the leaves (**Figure 8F**). The optimized 2^nd^ bud induction approach for transformation of SEE not only significantly (p<0.01) reduced chimera frequency (**Figure 8G**), but also significantly (p<0.01) improved overall non-chimeric transformation frequency to approximately 2% (**Figure 8H**).

### Improvement of rooting frequency

**Figure 9:**
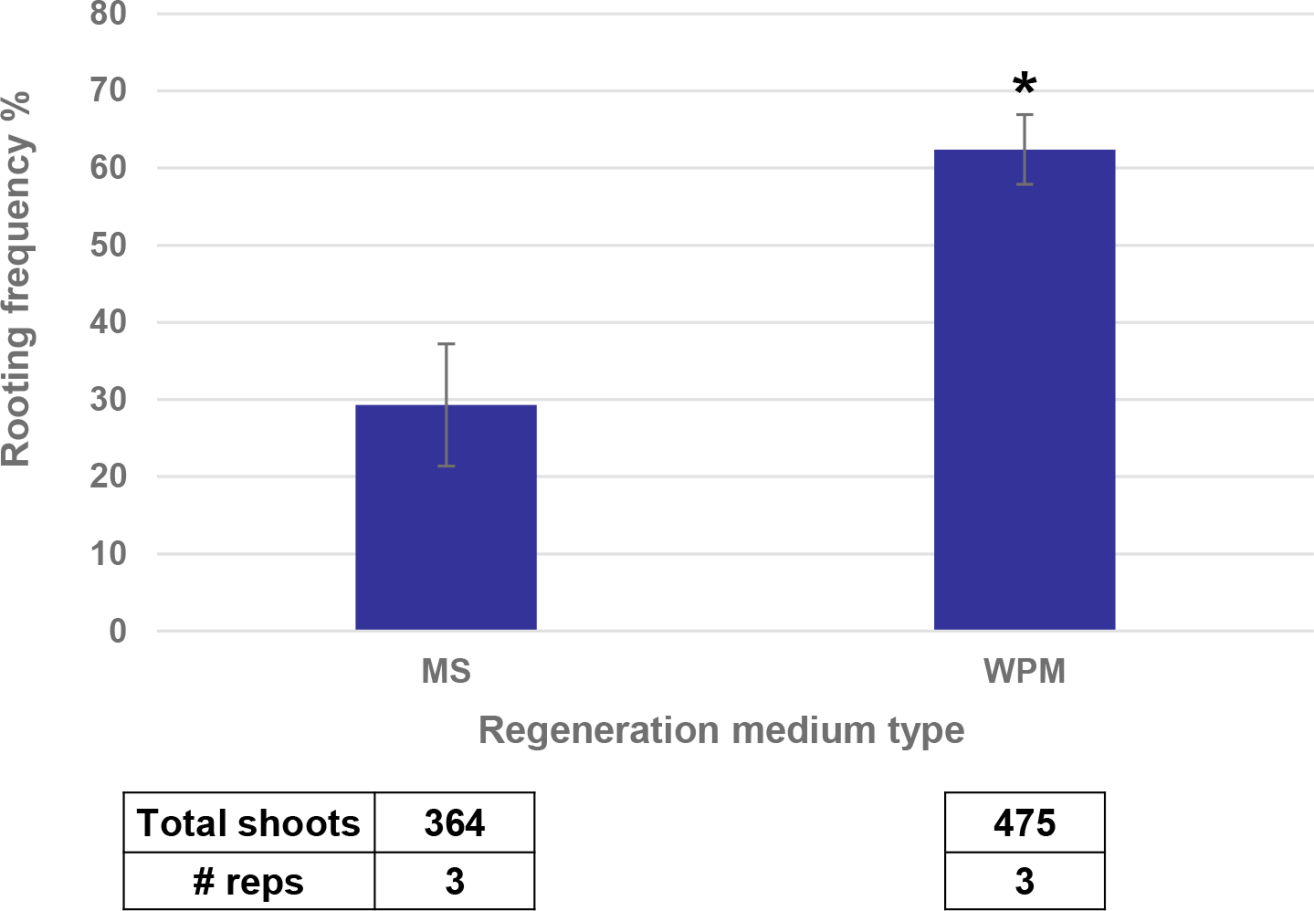
WPM medium increased the rooting frequency as compared to MS medium. * indicates significance at <0.05 level.

The last step to regenerate a whole plant from direct transformation of SEEs was the rooting process. Shoots produced through bud induction in the medium containing high cytokinin/auxin ratio, bud proliferation with different combination of cytokinin/auxin) and the regeneration in hormone free MS based medium had a low frequency of root differentiation. This made it necessary to transfer the shoots onto a rooting medium containing rooting hormone such as indole-3-butyric acid (IBA). However, this additional step of rooting was detrimental to plant health and caused abnormal phenotypes in the greenhouse, such as early flowering or tassel skeletonization. Substitution of MS based regeneration medium with WPM based regeneration medium led to the elongation of more transgenic buds resulting in better transformation frequency and a doubling in the rooting frequency (**Figure 9**).

## Discussion

This report demonstrates, for the first time, the transformation of a novel, easily available explants of the maize SEE through a multiple bud pathway to produce transgenic plants in a short timeline of 8 to 10 weeks. The successful transformation has been demonstrated with three different selectable makers, *cp4 epsps, aadA*, and *nptII* with non-chimeric transformation frequency up 2%. This system starts with mature seed that can be produced in large volumes in the field inexpensively. Seeds can be processed into SEEs in bulk and stored for long periods of time and can also be easily shipped and shared among researchers. This system has significant advantages over methods that rely on immature explants in terms of flexibility and scalability.

### Agrobacterium infection of relevant tissues

Unlike soybean and cotton SEEs, which mostly consist of intact embryos with an exposed shoot apical meristem, embryo axis and radicle (Martinell et al., 2002; Calabotta et al., 2013), the maize seed embryo explant used in this study is a partial embryo axis including part of a scutellar node and structures above the scutellar node including plumule and coleoptile (**Figures 1E, F**). The seed meristem region is covered by coleoptile and many layers of leaf primordia and therefore is not easily accessible to *Agrobacterium* for effective infection. One of our hypotheses is that *Agrobacterium* can access apical or leaf base meristem cells through the gaps between different layers of leaf primordia and that transient expression in coleoptile is positively corelated to *Agrobacterium* infection of relevant cells in apical meristem and leaf base meristem. Initial inoculation of freshly prepared SEEs with *Agrobacterium* harboring a *gusA* or *gfp* construct using protocols adapted from immature embryo transformation led to minimal transient expression on coleoptile. Vacuum infiltration and/or sonication steps that were routinely used during *Agrobacterium* inoculation in meristem transformation of dicot species, such as soybean and cotton (Ye et al., 2011; Chen et al., 2014) helped but did not show significant improvement. However, low concentration of potassium hydroxide (KOH) pre-treatment followed by sonication dramatically improved *Agrobacterium* infection. This could be due to that fact that part of the cell walls was dissolved by potassium hydroxide and the subsequent sonication-mediated micro-wounding of weakened cell walls and enabled the effective infection of coleoptile and potential meristematic cells in apical and leaf base meristems. Intuitively, additional physical wounding by removing part of the coleoptile would permit easy access of *Agrobacterium* to meristematic cells in the deep tissues. Nevertheless, the most significant improvement of *Agrobacterium* infection to the relevant cells was the centrifugation of explants during *Agrobacterium* inoculation. The leading hypothesis is that *Agrobacterium* cells were forced through tiny gaps between multiple layers of leaf primordia into the leaf base meristem or apical meristem cells and effectively contacted and transfected relevant tissues during the centrifugation. *Agrobacterium* could also infect apical or leaf base meristem cells through the mesocotyl area at the breaking point during meristem excision process. Pretreatment of immature embryos from maize and rice with centrifugation before *Agrobacterium* inoculation was shown to improve transformation previously (Hie et al., 2006).

### Efficient multiple bud induction from maize seed embryo explants

Following DNA delivery, SEEs consisting of apical meristems or leaf base-meristems would likely develop into chimeric plants if allowed to regenerate in hormone-free germination medium. Because most transgenic sectors would be restricted in size, the probability of a transformation event contributing to the germline without further manipulation would be very low. However, we were able to transform maize seed embryo explants and then regenerate transgenic plants by a brief induction of multiple buds through the manipulation of the cytokinin/auxin ratio followed by selection on hormone free media. When meristem explants containing transgenic sectors were cultured on medium containing various combination of plant hormones to enlarge the transgenic sectors, an increase in the likelihood of germline transmission through multiple bud pathway was observed. During the development of the protocol, we saw that multiple buds induced on high cytokinin and auxin medium for extended periods, led to difficulty in regeneration of shoots after transfer to hormone-free regeneration medium. Any shoots regenerated from those buds also were very difficult to root and rarely rooted plants had abnormal vegetative or reproductive phenotypes in the greenhouse (**Figures 3A–E**). Overcoming this challenge required the induction and, proliferation of multiple buds on media containing moderate level of cytokinin or moderate level of cytokinin/auxin ratios. Alternatively, buds could be induced on medium containing high level of cytokinin and high level of cytokinin/auxin ratio for a short period of time and multiplied on medium containing different combination of cytokinin/auxin ratio. These optimizations helped, leading to normal shoot and root elongation produced phenotypically normal transgenic events in the greenhouse. Other reports in literature show that multiple buds could be induced when shoot meristems were cultured on media containing cytokinin and high ratio of cytokinin/auxin whereas embryogenic callus could be induced on the medium containing auxin or low ratio of cytokinin/auxin (Zhong et al., 1992a; Zhong et al., 1992b; Lowe et al., 1995; Zhong et al., 1996; Li et al., 2002; Sairam et al., 2003; Lowe et al., 2016).

Culture of explants at 35°C for 4-7 days during the bud induction increased transgenic shoot regeneration as compared to culture of explants at 28°C in this study. We also found that culture of meristem explants at an elevated temperature for 3-6 days improved cotton meristem transformation previously (Chen et al., 2014). The culture of explants at an elevated temperature for a period of time before transfer to “normal” culture temperature in anther or microspore culture of many species, such as rapeseeds and flax might has been hypothesized to act as a signal to trigger the switch from the *in vivo* gametophytic pathway of microspores within anthers to the *in vitro* sporophytic pathway-leading to microspore embryogenesis (Keller and Armstrong, 1979, Chen et al., 1998). We hypothesized that short exposure of SEEs to the elevated temperature might signal meristem explants to transition from germination to bud differentiation and thereby enhance growth and differentiation of transformed cells in the meristem, leading to a higher transformation frequency. Wounding and other stress factors, including heat, has been shown to trigger cellular reprogramming to acquire pluripotency, leading to improved morphogenesis (Ikeuchi et al., 2020). Transcription factors may act as critical nodes to connect upstream stimuli, such as elevated temperature or wounding to downstream developmental decisions, such as bud induction and shoot regeneration.

### Choice of selectable markers in maize seed meristem transformation

A suitable selectable marker gene and selection reagent in the medium is critical to the maize SEEs transformation success. Three different selectable markers *cp4 epsps, aadA*, and *nptII* were successfully used to produce transgenic events in this study. As we expect direct regeneration of transgenic plantlets from meristematic cells through organogenesis pathway, living non-transgenic tissue may be required to support the transformed meristematic cells to form *de novo* meristems for shooting elongation. This was the case in soybean and cotton meristem explant transformation as the non-transgenic hypocotyl axis supports transgenic shoot elongation from transformed meristematic cells on the top of explants (Ye et al., 2011; Chen et al., 2014). Among these selective reagents, the compounds for *aadA* -spectinomycin and streptomycin) and *cp4 epsp* -glyphosate) are exclusively targeted to chloroplasts. The *aadA* marker with spectinomycin selection has been widely used for chloroplast transformation in dicot species (Maliga, 2004) as well as in our dicot SEEs transformation (Ye et al., 2011; Chen et al., 2014) because of the additional advantage of spectinomycin being able to be translocated to meristems as well (Pyke et al., 2000). However, wild type maize was found resistant to spectinomycin but sensitive to streptomycin. We use streptomycin for *aadA*-based selection and successfully regenerated transgenic plants. Since glyphosate is translocated primarily to metabolic sinks and kills meristematic tissues away from application sites (reviewed by Duke, 2018), we used sublethal level of glyphosate at 25 μM for initial soybean transgenic shoot elongation and 75 μM glyphosate for further selection since the non-transgenic hypocotyl was damaged during the sublethal selection (Ye et al., 2008). Similar methods were applied in maize SEE transformation to ensure non-transgenic living tissues supporting transformed meristematic cell differentiation in maize SEEs. Since introduction of glyphosate during 1st bud induction medium caused severe damage in explants, which often turned brown to black, we developed protocol with multiple bud induction media without selection initially to build up tolerance to glyphosate in co-cultured maize SEE, followed by selection on hormone-free rooting media for shoot elongation from transformed multiple buds. This led to the improvement of transformation frequency reported in this study.

### Steps to reduce the chimerism of transgenic plants

The first bud induction protocols resulted in up to 80% plants showing a visibly chimeric phenotype. Such a high frequency of chimerism in SEE-derived transgenic plants is not desirable in many biotechnology applications since it may impact germline transmission rates and may confound molecular analyses of primary transformants. The appearance of chimeric plants can be explained most plausibly by the multicellular origin of shoot organogenesis (Poethig, 1989; Zhu et al., 2007). Chimeras may also be a consequence of the protection of non-transgenic cells by the surrounding transformed cells (Park et al., 1998; Dominguez et al., 2004) or of the ineffectiveness of selective agents in species with an endogenous tolerance (Rakosy-Tican et al., 2007). Gould et al. (1991) transformed isolated shoot apices by *Agrobacterium* and observed high frequency of chimera. Zhong et al. (1996) transformed maize cultured shoot apices using microprojectile bombardment and observed chimeric clusters without selection pressure which required multiple steps of dissecting, dividing, and culturing of the surviving shoot tips on selection media until divided shoot-tip clumps uniformly survived under selection. The chimera issue was not revealed by using isolated maize meristems (Sairam et al., 2003) or meristem-derived multiple shoots (Cao et al., 2014), which might be due to small sample size and the non-phenotypical marker genes they used. The high chimera rate in our earlier experiments is likely due to the multiple bud induction without selection where the transformed cells together with surrounding cells co-develop into a chimeric bud. By shortening the no-selection bud induction time and applying selection in the second bud induction media, this allowed for the differentiation of non-chimera transformed buds through further cell division under selection, thereby, significantly (p<0.01) reducing the frequency of chimeric plants and improved the production of non-chimeric plants with high fertility.

In summary, we have demonstrated the development of a robust maize transformation system by transformation of dry excised meristem containing explants. This system significantly improved the feasibility, flexibility, and cost for many biotechnology applications in comparison with any conventional monocot transformation systems. In this system, we could easily handle tens thousands of seed meristem explants in a single experiment without the need of long-term planning as seed meristem explants were produced rather inexpensively from field-grown mature seeds and readily available from storage. We were able to produce events from many elite commercial lines that were found recalcitrant to transformation system *via* embryogenic callus, demonstrating genotype-flexible nature of this system. However, genotypic difference in transformation frequency still exists. We might be able to explore the complementarity of multiple bud induction and embryogenic systems to further improve transformation frequency and reduce chimera frequency. It would be very interesting to evaluate if a truly genotype-independent, high throughput transformation system would be possible through the combination of organogenesis based transformation of maize seed meristem explants along with over-expression of developmental regulators or growth regulating factors such as *BBM* and *WUS 2* (Lowe et al., 2016), *GRF5* and or GRF/GIF chimera (Kong et al., 2020; Debernardi et al., 2020, *WOX 5* (Wang et al., 2022) or LPT5 (Lian et al., 2022). The knowledge we have gained in this study may be applied in many other monocot species for both transformation and cell lineage of monocot meristem formation.

## Data availability statement

The original contributions presented in the study are included in the article/**Supplementary Material**. Further inquiries can be directed to the corresponding author.

## Author contributions

YC, XY, AS, and EW designed, conducted the experiments and analyzed the data. AR, ZV, LM, JK, SS, BM, and MS contributed to the data generation. YC, XY, AS, CA, MS, and DS wrote and/or edited the manuscript. All authors contributed to the article and approved the submitted version.

## Acknowledgments

We would like to thank former Monsanto and current Bayer colleagues K. Lockwood, G. Bean, S. Schroeder for *Agrobacterium* preparation; R. Byington, D. Warner, M. Irwin for media preparation; T. Emborg, J. Esser, J. Brewer, K. Janssen, L. Frank for molecular characterization; S. Hess, T. Van-Ert, M. Dickens, B. Reel for plant care in greenhouse and J. Arsenault, A. Chapman, Y. Wan, D. Duncan, V. Sidorov, R. Rode, D. Boyes for valuable advice and discussion; L. Gilbertson for help editing and revising the manuscript.

## Conflict of interest

YC, XY, AS, ZV, LM, SS, MS are current employees of Bayer CropScience, and EW, AR, JK, BM, CA, DS were former employees of Bayer CropScience or Monsanto Company, which was subsequently acquired by Bayer AG. Bayer CropScience develops and manufactures plant seeds and agricultural traits produced by conventional and biotechnology methods. Patent applications have been filed related to the subject matter of this manuscript and assigned to Monsanto or Bayer.

## Publisher’s note

All claims expressed in this article are solely those of the authors and do not necessarily represent those of their affiliated organizations, or those of the publisher, the editors and the reviewers. Any product that may be evaluated in this article, or claim that may be made by its manufacturer, is not guaranteed or endorsed by the publisher.

## Abbreviations

2,4-D, 2,4-dichlorophenoxyacetic acid; *aadA, *aminoglycoside adenylyltransferase gene; BAP, 6-benzylaminopurine; *cp4 epsps, Agrobacterium* CP4 5-enolpyruvulshikimate-3-phosphate synthase gene; GFP, green fluorescence protein; GUS, β-glucuronidase; IAA, indole acetic acid; NAA, naphthaleneacetic acid; *nptII, *neomycin phosphotransferase gene; TDZ, thidiazuron; SAM, shoot apical meristem; SEEs, seed embryo explants.

## References

[B1] Al-Abed D. Rudrabhatla S. Talla R. Goldman S. (2006). Split-seed: a new tool for maize researchers. Planta 223, 1355–1360. doi: 10.1007/s00425-006-0237-9 16489455

[B2] Altpeter F. Springer N. M. Bartley L. E. Blechl A. E. Brutnell T. P. Citovsky V.. (2016). Advancing crop transformation in the era of genome editing. Plant Cell 28, 1510–1520. doi: 10.1105/tpc.16.00196 27335450 PMC4981132

[B3] Armstrong T. Barton K. Crow L. J. Gilbertson L. A. Huang S. Huang Y.. (2010). Compositions for enhancing segregation of transgenes in plants, U.S. Patent No 7,858,370 (Washington, DC: U.S. Patent and Trademark Office). Available at: https://patents.google.com/patent/US7858370B2/en

[B4] Calabotta B. J. Deppermann K. L. Dersch E. Heise J. D. Koestel A. R. Ludwig C. L.. (2013). Preparation and use of plant embryo explants for transformation, US Patent US8362317B2. (Washington, DC: U.S. Patent and Trademark Office). Available at: https://patents.google.com/patent/US8362317B2/en?oq=US8362317B2

[B5] Cao S. L. Masilamany P. Li W. B. Pauls K. P. (2014). *Agrobacterium tumefaciens*-mediated transformation of corn (*Zea mays* l.) multiple shoots. Biotechnol. Biotechnol. Equip. 28, 208–216. doi: 10.1080/13102818.2014.907654 26019506 PMC4433900

[B6] Chen Y. Lange A. Vaghchhipawala Z. Ye X. Saltarikos A. (2020). Direct germline transformation of cotton meristem explants with no selection. Front. Plant Sci. 11. doi: 10.3389/fpls.2020.575283 PMC7543975 33072151

[B7] Chen Y. Rivlin A. Lange A. Ye X. Vaghchhipawala Z. Eisinger E.. (2014). High throughput *Agrobacterium tumefaciens*-mediated germline transformation of mechanically isolated meristem explants of cotton (*Gossypium hirsutum* l.). Plant Cell Rep. 33, 153–164. doi: 10.1007/s00299-013-1519-x 24129847

[B8] Chen Y. Kenaschuk E. O. Procunier D. J. (1998). Plant regeneration from anther culture in canadian cultivars of flax (Linum usitatissimum l.). Euphytica 102, 183–189.

[B9] Debernardi J. M. Tricoli D. M. Ercoli M. F. Hayta S. Ronald P. Palatnik J. F.. (2020). A GRF–GIF chimeric protein improves the regeneration efficiency of transgenic plants. Nat. Biotechnol. 38, 1274–1279. doi: 10.1038/s41587-020-0703-0 33046875 PMC7642171

[B10] Dellaporta S. L. Wood J. Hicks J. B. (1983). A plant DNA minipreparation: version II. Plant Mol. Biol. Rep. 1, 19–21. doi: 10.1007/BF02712670

[B11] Dominguez A. Cervera M. Perez R. M. Romero J. Fagoaga C. Cubero J. (2004). Characterization of regenerants obtained under selective conditions after *Agrobacterium*-mediated transformation of citrus explants reveals production of silenced and chimeric plants at unexpected high frequencies. Mol. Breed. 14, 171–183. doi: 10.1023/B:MOLB.0000038005.73265.61

[B12] Duke S. O. (2018). The history and current status of glyphosate. Pest Manage. Sci. 74, 1027–1034. doi: 10.1002/ps.4652 28643882

[B13] Fromm M. Morrish F. Armstrong C. Williams R. Thomas J. Klein T. (1990). Inheritance and expression of chimeric genes in the progeny of transgenic maize plants. Bio/technol. 8, 833–839. doi: 10.1038/nbt0990-833 1366794

[B14] Gordon-Kamm W. J. Spencer T. M. Mangano M. L. Adams T. R. Daines R. J. Start W. G.. (1990). Transformation of maize cells and regeneration of fertile transgenic plants. Plant Cell 2, 603–618. doi: 10.2307/3869124 12354967 PMC159915

[B15] Gould J. Devey M. Hasegawa O. Ulian E. Peterson G. Smith R. (1991). Transformation of *Zea mays* l. using *Agrobacterium tumefaciens* and the shoot apex. J. Plant Physiol. 95, 426–434. doi: 10.1104/pp.95.2.426 PMC1077548 16668001

[B16] Hie i Y. Ishida Y. Kasaoka K. Komari T. (2006). Improved frequency of transformation in rice and maize by treatment of immature embryos with centrifugation and heat prior to infection with *Agrobacterium tumefaciens.* plant cell tiss. Organ Cult. 87, 233–243. doi: 10.1007/s11240-006-9157-4

[B17] Huang X. Wei Z. (2004). High frequency plant regeneration through callus initiation from mature embryos of maize (*Zea mays* l.). Plant Cell Rep. 22, 793–800. doi: 10.1007/s00299-003-0748-9 15022014

[B18] Ikeuchi M. Rymen B. Sugimoto K. (2020). How do plants transduce wound signals to induce tissue repair and organ regeneration? Curr. Opin. Plant Biol. 57, 72–77. doi: 10.1016/j.pbi.2020.06.007 32738736

[B19] Ishida Y. Satto H. Ohta S. Hiei Y. Komari T. Kumashiro T. (1996). High efficiency transformation of maize (*Zea mays* l.) mediated by *Agrobacterium tumefaciens.* nat. Biotech. 14, 745–750. doi: 10.1038/nbt0696-745 9630983

[B20] Jefferson R. A. (1989). The GUS reporter gene system. Nature 342 (6251), 837–838. doi: 10.1038/342837a0 2689886

[B21] Kausch A. P. Wang K. Kaeppler H. F. Gordon-Kamm W. (2021). Maize transformation: history, progress, and perspectives. Mol. Breed. 41, 1–36. doi: 10.1007/s11032-021-01225-0 PMC10236110 37309443

[B22] Keller W. A. Armstrong K. C. (1979). Stimulation of embryogenesis and haploid production in brassica campestris anther cultures by elevated temperature treatments. Theor. Appl. Genet. 55, 65–67. doi: 10.1007/BF00285191 24306485

[B23] Kiesselbach T. A. (1949). The structure and reproduction of corn. Res. bulletin: Bull. Agric. 161, 96.

[B24] Kong J. Martin-Ortigosa S. Finer J. Orchard N. Gunadi A. Batts L. A.. (2020). Overexpression of the transcriptional facto GROWTH-REGULATING FACTORS5 improves transformation of dicot and monocot species. Front. Plant Sci. 11, 572319. 33154762 10.3389/fpls.2020.572319 PMC7585916

[B25] Lian Z. Nguyen C. Liu L. Wang G. Chen J. Wang S.. (2022). Application of developmental regulators to improve *in planta* or *in vitro* transformation in plants. Plant Biotechnol. J. 8, 1622–1635. doi: 10.1111/pbi.13837 PMC9342618 35524453

[B26] Li W. Masilamany P. Kasha K. Pauls P. (2002). Development, tissue culture and genotypic factors affecting plant regeneration from shoot apical meristems of germinating *Zea mays* l. seedlings. In Vitro Cell. Dev. Biol.-Plant 38, 285–292. doi: 10.1079/IVP2002291

[B27] Lowe K. Bowen B. Hoerster G. Ross M. Bond D. Pierce D.. (1995). Germline transformation of maize following manipulation of chimeric shoot meristem. Bio/technol 13, 677–682. doi: 10.1038/nbt0795-677

[B28] Lowe K. Wu E. Wang N. Hoerster G. Hastings C. Cho M. J.. (2016). Morphogenic regulators baby boom and wuschel improve monocot transformation. Plant Cell 28, 1998–2015. doi: 10.1105/tpc.16.00124 27600536 PMC5059793

[B29] Maliga P. (2004). Plastid transformation in higher plants. Annu. Rev. Plant Biol. 55, 289. doi: 10.1146/annurev.arplant.55.031903.141633 15377222

[B30] Martinell B. Julson L. S. Emler C. A. Huang Y. McCabe D. E. Williams E. J. (2002). Soybean agrobacterium transformation method, US Patent # US6384301. (Washington, DC: U.S. Patent and Trademark Office). Available at: https://patents.google.com/patent/US6384301B1/en?oq=US6384301

[B31] Martinell B. Petersen. M. Somers. D. Wan. Y. Williams E. Ye X. (2013). Methods for plant transformation using spectinomycin selection, US Patent # US8466345B2. (Washington, DC: U.S. Patent and Trademark Office). Available at: https://patents.google.com/patent/US8466345B2/en?oq=US8466345B2

[B32] McCabe D. E. Martinell B. J. (1993). Transformation of elite cotton cultivars *via* particle bombardment of meristems. Bio/Technol. 11, 596–598. doi: 10.1007/BF02319006

[B33] McCabe D. E. Swain W. F. Martinell B. J. Christou P. (1988). Stable transformation of soybean (*Glycine max*) by particle acceleration. Bio/Technol. 6, 923–926. doi: 10.1038/nbt0888-923

[B34] Park S. H. Rose S. C. Zapata C. Srivatanakul M. Smith R. H. (1998). Cross-protection and selectable marker genes in plant transformation. In Vitro Cell. Dev. Biol.-Plant 34, 117–121. doi: 10.1007/BF02822775

[B35] Poethig S. (1989). Genetic mosaics and cell lineage analysis in plants. Trends Genet. 5, 273–277. doi: 10.1016/0168-9525(89)90101-7 2686117

[B36] Pyke K. Zubko M. K. Day A. (2000). Marking cell layers with spectinomycin provides a new tool for monitoring cell fate during leaf development. J. Exp. Bot. 51, 1713–1720. doi: 10.1093/jexbot/51.351.1713 11053461

[B37] Rakosy-Tican E. Aurori C. M. Dijkstra C. Thieme R. Aurori A. Davey M. R. (2007). The usefulness of the *gfp* reporter gene for monitoring *Agrobacterium*-mediated transformation of potato dihaploid and tetraploid genotypes. Plant Cell Rep. 26, 661–671. doi: 10.1007/s00299-006-0273-8 17165042

[B38] Sairam R. V. Parani M. Franklin G. Lifeng Z. Smith B. MacDougall J.. (2003). Shoot meristem: an ideal explant for *Zea mays* l. transformation. Genome 46, 323–329. doi: 10.1139/g02-120 12723048

[B39] SAS/STAT User’s Guide (1989). Version 6, 4th edition Vol. Vol 2 (Cary, NC: SAS Institute Inc.).

[B40] Sidorov V. Gilbertson L. Addae P. Duncan D. (2006). Agrobacterium-mediated transformation of seedling-derived maize callus. Plant Cell Rep. 25 (4), 320–328. doi: 10.1007/s00299-005-0058-5 16252091

[B41] Songstad D. D. Armstrong C. L. Petersen W. L. Hariston B. Hinchee M. A. W. (1996). Production of transgenic maize plants and progeny by bombardment of Hi-II immature embryos. In Vitro Cell. Dev. Biol.- Plant 32, 179–183. doi: 10.1007/BF02822763

[B42] Sticklen M. Oraby H. (2005). Shoot apical meristem: A sustainable explants for genetic transformation of cereal crops. In Vitro Cell. Dev. Biol.- Plant 41, 187–200. doi: 10.1079/IVP2004616

[B43] Vaghchhipawala Z. Radke S. Nagy E. Russell M. L. Johnson S. Gelvin S. B.. (2018). RepB c-terminus mutation of a pRi-repABC binary vector affects plasmid copy number in agrobacterium and transgene copy number in plants. PloS One 13 (11), e0200972. doi: 10.1371/journal.pone.0200972 30412579 PMC6226153

[B44] Walters D. Vetsch C. Potts D. Lundquist R. (1992). Transformation and inheritance of a hygromycin phosphotransferase gene in maize plants. Plant Mol. Biol. 18, 189–200. doi: 10.1007/BF00034948 1310057

[B45] Wang A. S. (1987). Callus induction and plant regeneration from maize mature embryos. Plant Cell Rep. 6, 360–362. doi: 10.1007/BF00269560 24248845

[B46] Wang K. Shi L. Liang X. Zhao P. Wang W. Liu J.. (2022). The gene TaWOX5 overcomes genotype dependency in wheat genetic transformation. Nat. Plants 8, 110–117. doi: 10.1038/s41477-022-01173-3 35027699

[B47] Ye X. Chen Y. Wan Y. Hong Y. J. Ruebelt M. C. Gilbertson L. A. (2016). Constitutive expression of the *tzs* gene from *Agrobacterium tumefaciens* virG mutant strains is responsible for improved transgenic plant regeneration in cotton meristem transformation. Plant Cell Rep. 35, 601–611. doi: 10.1007/s00299-015-1906-6 26650837

[B48] Ye X. Williams E. J. Shen J. Esser J. A. Nichols A. M. Petersen M. W.. (2008). Plant development inhibitory genes in binary vector backbone improve quality event efficiency in soybean transformation. Transgenic Res. 17, 827–838. doi: 10.1007/s11248-008-9169-4 18253857

[B49] Ye X. Williams E. J. Shen J. Johnson S. Lowe B. Radke S.. (2011). Enhanced production of single copy backbone-free transgenic plants in multiple crop species using binary vectors with a pRi replication origin in *Agrobacterium tumefaciens* . Transgenic Res. 20, 773–786. doi: 10.1007/s11248-010-9458-6 21042934

[B50] Zhang S. Williams C. R. Lemaux P. (2002). Transformation of recalcitrant maize inbreds using *in vitro* shoot meristematic cultures induced from germinated seedlings. Plant Cell Rep. 21, 263–270. doi: 10.1007/s00299-002-0513-5

[B51] Zhong H. Srinivasan C. Sticklen M. B. (1992a). *In-vitro* morphogenesis of corn (*Zea mays* l.): I. differentiation of multiple shoot clumps and somatic embryos from shoot tips. Planta 187, 483–489. doi: 10.1007/BF00199966 24178142

[B52] Zhong H. Srinivasan C. Sticklen M. B. (1992b). In-vitro morphogenesis of corn (*Zea mays* l.): II. differentiation of ear and tassel clusters from cultured shoot apices and immature inflorescences. Planta 187, 490–497. doi: 10.1007/BF00199967 24178143

[B53] Zhong H. Sun B. Warkentin D. Zhang S. Wu R. Wu T.. (1996). The competence of maize shoot meristems for integrative transformation and inherited expression of transgenes. Plant Physiol. 110, 1097–1107. doi: 10.1104/pp.110.4.1097 12226244 PMC160889

[B54] Zhu X. Y. Zhao M. Ma S. Ge Y. M. Zhang M. F. Chen L. P. (2007). Induction and origin of adventitious shoots from chimeras of *Brassica juncea* and *Brassica oleracea* . Plant Cell Rep. 26, 1727–1732. doi: 10.1007/s00299-007-0398-4 17622536

