# Supplementary material for: Commercial scale genetic transformation of mature seed embryo explants in maize
Source: Front Plant Sci. 2022 Nov 29;13:1056190. doi: 10.3389/fpls.2022.1056190 (PMC9745677; doi:10.3389/fpls.2022.1056190)
Supplement: Supplementary file 1 [file DataSheet_1.docx]

# Large scale genetic transformation of mature seed embryo explants in maize

Xudong Yeꝉ^1^, Ashok Shrawatꝉ^1^, Edward Williams^2^, Anatoly Rivlin^2^, Zarir Vaghchhipawala^1^, Lorena Moeller^1^, Jennifer Kumpf^3^, Shubha Subbarao^1^, Brian Martinell^1^, Charles Armstrong^1^, M. Annie Saltarikos^1^, David Somers^3^, Yurong Chenꝉ^1, 4^

ꝉThese authors contributed equally to this work

1Plant Biotechnology, Bayer Crop Science,700 Chesterfield Pkwy, W. St. Louis, MO, 63017, USA

2 Agracetus Campus, Monsanto Company, 8520 University Green, P.O. Box 620999, Middleton, WI, 53562

3 Mystic Research, Monsanto Company, 62 Maritime Drive, Mystic, CT 06355, USA

4 To whom correspondence should be addressed

Address correspondence to

Dr. Yurong Chen

Plant Biotechnology

Bayer Crop Science,

700 Chesterfield Pkwy, W. St. Louis, MO 63017

[yurong.chen@bayer.com](mailto:yurong.chen@bayer.com)


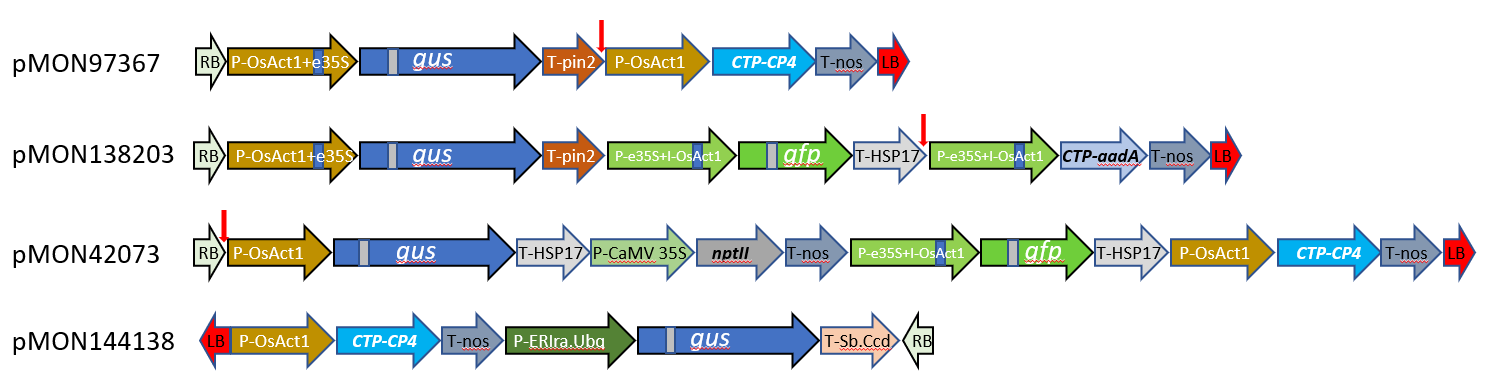


**Supplemental Figure S1:** Constructs used for maize SEEs transformation development. Vertical arrows indicate *HindIII* cut for Southern blot. Note: pMON138210 is identical to pMON138203 except that the *aadA* coding sequence is codon optimized for monocot expression. pMON138210 is identical to pMON138203, except for the codon optimization of *aadA*. **P-OsAct1+e35S**: rice actin 1 promoter with CaMV enhancer sequence; ***gus****: gusA gene with intron (*Vancanneyt et al., 1990); ***T-pin2:*** Potato proteinase inhibitor II terminator (GenBank accession X04118)***;***  ***CP4****: cp4 epsps gene from Agrobacterium* CP4 strain encoding for 5-enolpyruvulshikimate-3-phosphate synthase; ***T-nos****: Agrobacterium nos* transcription terminator (Depicker et al. 1982); **P-ERIra.Ubq**: *Tripidium ravennae* ubiquitin promoter (GenBank MH026095); **P-eCaMV35S**: enhanced CaMV 35S promoter; ***aadA***: aminoglycoside (3'') (9) adenylyltransferase gene confers resistance to the aminoglycosides spectinomycin; **T-HSP17**: wheat heat shock protein terminator (GenBank accession X13431); ***gfp****:* green fluorescent protein gene (Pang et al., 1996); ***nptII***: neomycin phosphotransferase gene; **T-Sb.Ccd**: *Sorghum bicolor* cortical cell-delineating protein terminator (GenBank accession XM_002450283.2)

**References:**

1.Depicker A, Stachel S, Dhaese P, Zambryski P, Goodman HM. (1982) Nopaline synthase: transcript mapping and DNA sequence. J Mol Appl Genet. 1:561-73. (no DOI #)

2.Pang SZ, DeBoer DL, Wan Y, Ye G, Layton JG, Neher MK, Armstrong CL, Fry JE, Hinchee MA, Fromm ME. (1996) An improved green fluorescent protein gene as a vital marker in plants. Plant Physiol. 112: 893–900. https://doi:10.1104/pp.112.3.893

3.Vancanneyt G, Schmidt R, O’Connor-Sanchez A, Willmitzer L, Rocha-Sosa M (1990) Construction of an intron-containing marker gene: splicing of the intron in transgenic plants and its use in monitoring early events in *Agrobacterium*-mediated plant transformation. Mol Genet Genomics. 220:245–2503. <https://doi.org/10.1007/BF00260489>


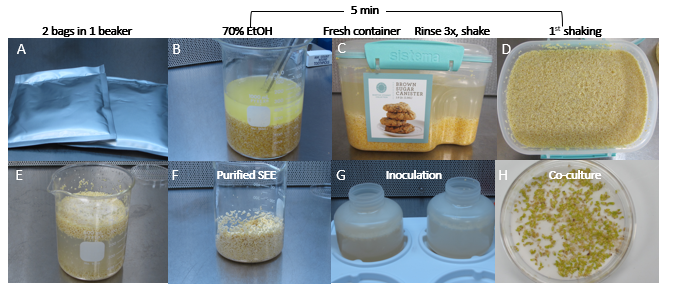


**Supplemental Figure S2:** Purification of maize SEE by water floatation. **A**) Ground maize seed particles sealed in a Mylar bag and stored at -20 or -80 ^o^C; **B**) Pour two bags (20,000 SEEs each) into 1 liter beaker, add 70% ethanol to cover the crushed corn particles for about 4 min, pour off the ethanol and rinsed with 500 ml sterilized water 3 times; **C**) Transfer into the canister, add 1 liter water, close the lid with snap lock, shake 1 to 3 times to float SEEs; D) SEE float on surface; E) Scoop the SEE into a 500 ml beaker with 500 ml water, rinse with water 3 times; **F**) Rinsed SEEs ready for inoculation; **G**) Transfer SEEs into 500 ml conical centrifuge tube with 300 to 500 ml *Agrobacterium* suspension; centrifuge at 291 xg for 30 mins, remove *Agrobacterium* suspension completely after centrifuging and pouring into a Plantcon lid; **H**) Spread 1 layer of inoculated *Agrobacterium* SEEs on a filter paper for co-culture.


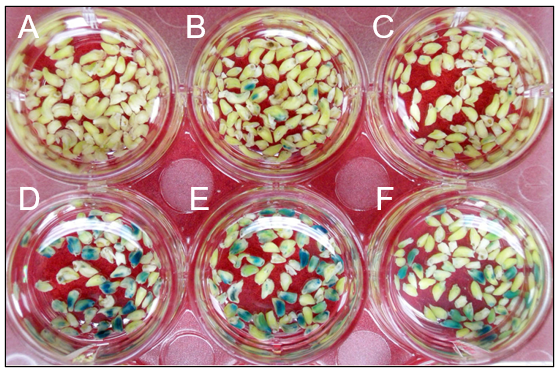


**Supplemental Figure S3.**  T-DNA delivery into relevant tissues of maize SEEs by KOH pre-treatment and sonication during inoculation. 2 mM KOH pretreatment of SEE before *Agrobacterium* inoculation, followed by vacuum and/or sonication. **A**) control, no *Agrobacterium*, vacuum twice, sonication 1 min; **B**) with *Agrobacterium*, vacuum 1x; **C**) with *Agrobacterium*, vacuum 2x; **D**) with *Agrobacterium*, sonication 1 min; **E**) with *Agrobacterium*, vacuum 1x, and followed by sonication 1 min; **F**) with *Agrobacterium*, vacuum 2x, and followed by sonication 1 min. pMON42073 (Figure S1) was used for these experiments.

**Supplemental Figure S4.** The effect of centrifugation, sonication, and temperature on T-DNA delivery to maize SEEs during inoculation with *Agrobacterium*. The negative control treatment (center) was not inoculated. Four treatments (on the left) were sonicated for 1 minute at 45 kHz, whereas four treatments (on the left) were not. Additional treatments (as labeled) were centrifugation at 291 xg, or no centrifugation and temperatures of 4⁰C or 23⁰C. Expression of gusA was measured as an activity in a MUG fluorometric assay for quantitative analysis of beta-glucuronidase (GUS) activity.

**Supplemental Figure S5.** *Agrobacterium* infection of SEEs of multiple germplasms under different inoculation conditions. (**A**) to (**C)** Infection of inbred line BPL1, BPL2 and LH244 inoculated with AB32 / pMON138203 without centrifugation; (**D**) to (**F**) Infection of inbred lines BPL1, BPL2 and LH244 inoculated with AB32 / pMON138203 with centrifugation at 291 g.

**Supplemental Figure S6**. The effect of co-culture medium on transient expression. (**A**) Co-culture medium 1595; (**B**) Co-culture medium 1595 with 5 mg/l 2,4-D; (**C**) Co-culture medium 1484; (**D**) Co-culture medium 1273.


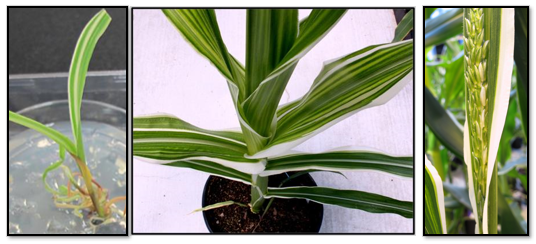


**Supplemental Figure S7.** Examples of phenotypic chimerism of primary transformed plants selected on glyphosate. **From left to right**: striped plantlet from tissue culture, striped leaves from plant in soil, striped leaf and tassel from plant in greenhouse.

**Supplemental Table S1**. Transmission of transgenes into the next generation in maize SEE transformation

| Event | Vector | Selectable marker | Estimated GUS copy | Estimated CP4 copy | Pollen staining | GUS+ : GUS in F1 progeny | Exp. ratio | Chi-square value |
| --- | --- | --- | --- | --- | --- | --- | --- | --- |
| 2 | pMON 42073 | npt II | 1 | 1 | ND | 21:19 | 1:1 | 0.03 |
| 3 | pMON 97367 | cp4 | 2 | 2 | ND | 17:19 | 1:1 | 0.03 |
| 4 | pMON 97367 | cp4 | 4 | 2 | ND | 8:11 | 1:1 | 0.21 |
| 5 | pMON 97367 | cp4 | 5 | 2 | segregation | 8:15 | 1:1 | 1.57 |
| 6 | pMON 97367 | cp4 | >4 | 4 | segregation | 25:23 | 1:1 | 0.02 |
| 7 | pMON 97367 | cp4 | 8 | >4 | negative | 1:47 | 1:1 | 42.2* |
| 8 | pMON 97367 | cp4 | 4 | 2 | ND | 15:22 | 1:1 | 0.97 |
| 9 | pMON 97367 | cp4 | 3 | 1 | segregation | 23:19 | 1:1 | 0.21 |
| 10 | pMON 97367 | cp4 | >8 | >6 | ND | 10:1 | 3:1 | 0.76 |
| 12 | pMON 97367 | cp4 | 1 | 1 | segregation | 28:46 | 1:1 | 3.90* |
| 13 | pMON 97367 | cp4 | 2 | 1 | segregation | 23:12 | 1:1 | 2.86 |
| 15 | pMON  138210 | aadA | 1 | 0 | segregation | 19:20 | 1:1 | 0.00 |
